# Supplementary material for: H2-driven biocatalysis for flavin-dependent ene-reduction in a continuous closed-loop flow system utilizing H2 from water electrolysis
Source: Commun Chem. 2024 Sep 7;7:200. doi: 10.1038/s42004-024-01288-y (PMC11380674; doi:10.1038/s42004-024-01288-y)
Supplement: Supplementary file 2 — Supplementary Information [file 42004_2024_1288_MOESM2_ESM.pdf]

Supporting Information for:

H_2-driven biocatalysis for flavin-dependent ene-reduction in a continuous closed-loop flow system utilizing H_2 from water electrolysis

Guiyeoul Lim [1], Donato Calabrese [1], Allison Wolder [2], Paul R. F. Cordero [1], Dörte Rother [1,3], Florian Mulks [4], Caroline E. Paul [2], Lars Lauterbach [1]

[1] Institute of Applied Microbiology - iAMB
RWTH Aachen University, 52074 Aachen (Germany)

[2] Biocatalysis section, Department Biotechnology
Delft University of Technology, 2629HZ Delft (The Netherlands)

[3] Institute for Bio-and Geosciences 1: Biotechnology
Forschungzentrum Jülich GmbH, 52425 Jülich (Germany)

[4] Institute of Organic Chemistry - iOC
RWTH Aachen University, 52074 Aachen (Germany)

Table of Contents

1. Chemicals and Materials 2

2. Enzyme parameters 2

2.1 SH, SH-Tactin activity assay 2

2.2 *Ts*OYE, *Ts*OYE-EziG activity assay 2

2.3 Calculation of enzyme immobilization parameters for corresponding carriers 3

2.4 Characterization of immobilized biocatalysts 4

3. Continuous flow setup 5

3.1 Immobilization of biocatalysts, biotransformation unit preparation 6

3.2 Electro/-H_2 driven biocatalysis (ketoisophorone as substrate, reusability of the biocatalyst experiments) 6

3.3 Electro/-H_2 driven biocatalysis (cyclohexenone as substrate) 6

3.4 Electro/-H_2 driven biocatalysis ((*R*)-carvone, (*S*)-carvone as substrate) 6

3.5 Electro/-H_2 driven biocatalysis (upscale reaction 185 mL, ketoisophorone as substrate) 7

4. H_2 transfer rate 7

5. *Ts*OYE immobilization on different EziG beads 8

6. Enzymatic membrane reactor (EMR) 9

7. Electro-driven biotransformation with immobilized enzymes 9

8. Reusability of the immobilized enzymes 10

9. Total turnover number for reused immobilized biocatalysts 10

10. Conversion rate during upscaled reaction 11

11. Comparison with literature 11

12. Faradaic efficiency 12

13. Environmental impact 12

14. GC analyses 13

14.1 GC-FID 13

14.2 GC-FID Chiral 20

14.3 GC-MS 24

15. ^{1}H Nuclear magnetic resonance spectra of the isolated levodione 28

Supplementary References 30

Supplementary Methods

1. Chemicals and Materials

Ethyl acetate, flavin mononucleotide (FMN), catalase, substrates (cyclohexenone, ketoisophorone, (*R*)-carvone, (*S*)-carvone) and products (cyclohexanone, mixture of (+)-dihydrocarvone) for standards were purchased from Sigma-Aldrich (Germany). (6*R*)-Levodione was kindly provided by Adrie Straathof (TU Delft). Buffer components were bought from SERVA (Germany). Chemicals for media preparations were obtained from Carl Roth GmbH (Germany). Peristaltic pump (Reglo ICC) was purchased from IDEX (USA). Portable spectrophotometer (Model Genova Bio, Jenway) was bought from Cole-Parmer (USA). O_2 adhesive spot sensor was purchased from PreSens (Germany). H_2 sensor was purchased from Unisense (Denmark). Mini flow cuvette was obtained from Hellma analytics (Germany). PEM electrolyzer (E206, 65) was obtained from H-TEC education. Teflon (OD 2 mm, ID 1.5 mm, length 2 m), tubing was bought from CS-Chromatographie Service GmbH (Germany). Fluran® was purchased from VWR (Germany). Streptactin XT 4Flow resin (20 mL) was bought from IBA-life sciences (Germany) while EziG carriers were kindly provided by EnginZyme. C 10/10 Column was purchased from Cytiva (USA). Heating cabinet was bought from HARTMANN.

2. Enzyme parameters

2.1 SH, SH-Tactin activity assay

(1) SH activity assay

SH activity assay by H_2-dependent reduction of FMN was performed spectrophotometrically as described in (Al-Shameri et al. 2020).

(2) SH-Tactin activity assay

500 mg of Strep-Tactin XT 4Flow resin was loaded with 1.66 mg SH to achieve 3.3 mg g^{-1} carrier loading. The immobilized SH-Tactin was used to perform H_2-dependent reduction of FMN. Activity measurements were performed spectrophotometrically using Agilent Technologies Cary 60 UV-Vis spectrophotometer by monitoring the absorbance decrease of FMN at 500 nm in 2 mL cuvettes after purging with H_2. After addition of SH-Tactin, the cuvette was shaken before measurement in the spectrophotometer. The activity of SH-Tactin was measured in 50 mM Tris-HCl pH 8, at 30 °C. The specific activities were calculated using the extinction coefficient of FMN at 500 nm. The extinction coefficient of FMN and FAD at 500 nm $\varepsilon = 2.55\ mM^{-1}cm^{-1}$.

2.2 *Ts*OYE, *Ts*OYE-EziG activity assay

(1) *Ts*OYE activity assay

Activity measurements of His-tagged *Ts*OYE were performed spectrophotometrically using Agilent Technologies Cary 60 UV-Vis spectrophotometer by monitoring the NADPH absorbance decrease at 365 nm in 2 mL cuvette with 1 mM NADPH and 25 mM cyclohexenone. After addition of *Ts*OYE (6 µg), the cuvette was shaken before measurement in the spectrophotometer. The activity of *Ts*OYE was measured in 50 mM Tris-HCl pH 7.5, at 30 °C. The specific activities were calculated using the extinction coefficient of NADPH at 365 nm. The extinction coefficient of NADH and NADPH at 365 nm $\varepsilon = 3.3\ mM^{-1}cm^{-1}$.

(2) *Ts*OYE-EziG activity assay

50 mg of EziG beads (Amber) was first loaded with 0.72 mg *Ts*OYE to achieve 14.4 mg g^{-1} carrier loading. This was shaken overnight and used for perform NADPH-dependent reduction of cyclohexenone as described in 2.2- (1). *Ts*OYE-EziG was added by the amount of 0.416 mg for the activity assay. NADPH was chosen instead of $FMNH_2$, due to its ease of control with stoichiometric addition.

(3) His-tagged *Ts*OYE sequences

MGSSHHHHHHSSGLVPRGSHMALLFTPLELGGLRLKNRLAMSPMCQYSATLEGEVTDWHLLHYPTRALGGVGLILVEATAVEPLGRISPYDL
GIWSEDHLPGLKELARRIREAGAVPGIQLAHAGRKAGTARPWEGGKPLGWRVVGPSPIPFDEGYPVPEPLDEAGMERILQAFVEGARRALRA
GFQVIELHMAHGYLLSSFLSPLSNQRTDAYGGSLENRMRFPLQVAQAVREVVPRELPLFVRVSATDWGEGGWSLEDTLAFARRLKELGVDLL
DCSSGGVVLRVRIPLAPGFQVPFADAVRKRVGLRTGAVGLITTPEQAETLLQAGSADLVLLGRVLLRDPYFPLRAAKALGVAPEVPPQYQRGF*

ATGGGCAGCAGCCATCATCATCATCATCACAGCAGCGGCCTGGTGCCGCGCGGCAGCCATATGGCCTTGCTCTTCACCCCCCTGGAACTC
GGCGGCCTCCGGCTGAAAAACCGCCTGGCCATGTCCCCCATGTGCCAGTACTCCGCCACCTTGGAGGGAGAGGTAACCGACTGGCACCT
CCTCCACTACCCCACGCGGGCCCTTGGGGGCGTGGGGCTCATTCTGGTGGAGGCCACCGCCGTGGAACCTTTGGGCCGTATCAGCCCCT
ATGACCTGGGCATCTGGTCGGAGGATCACCTTCCGGGCCTGAAGGAGCTCGCCCGGAGGATCCGGGAAGCTGGAGCGGTGCCGGGGA
TCCAGCTGGCCCACGCCGGGCGCAAGGCGGGGACCGCCAGGCCCTGGGAAGGGGGAAAGCCCCTGGGCTGGCGGGTGGTGGGGCCA
AGCCCCATTCCCTTTGACGAGGGCTACCCGGTACCCGAACCCCTGGACGAAGCAGGGATGGAGCGCATCCTCCAGGCCTTCGTGGAAG
GAGCCAGACGTGCCCTTAGGGCAGGCTTTCAGGTGATCGAGCTCCACATGGCCCATGGCTACCTCCTTTCCTCCTTCCTCTCCCCCCTTTC
CAACCAGCGCACCGACGCCTACGGGGGAAGCCTGGAAAACCGCATGCGCTTTCCCCTCCAGGTGGCCCAGGCAGTGCGGGAGGTGGT
GCCCAGGGAGCTTCCCCTTTTCGTGCGGGTCTCCGCCACGGACTGGGGGGAAGGAGGATGGAGCCTCGAGGACACCCTGGCCTTCGCC
CGGAGGCTTAAGGAGCTGGGGGTGGACCTTTTGGACTGCTCCTCGGGCGGGGTGGTGCTCAGGGTGCGGATTCCCCTGGCCCCGGGCT
TTCAGGTGCCCTTCGCCGACGCCGTGCGCAAGAGGGTGGGCCTGCGAACGGGAGCCGTGGGCCTCATCACCACCCCCGAGCAGGCGG
AAACCCTCCTGCAGGCGGGAAGCGCCGATCTGGTGCTTCTGGGCCGGGTTCTCCTCAGGGACCCCTACTTCCCCTTACGGGCTGCCAAG
GCCTTGGGCGTGGCCCCGGAGGTACCCCCCCAGTACCAAAGGGGGTTTTAG

2.3 Calculation of enzyme immobilization parameters for corresponding carriers

To determine the applicability of the carriers in immobilizing the biocatalysts, different parameters were assessed.

(1) Immobilization yield

Percentage of total enzyme immobilized on the carrier; the yield was assessed by quantifying the protein content in the washing solution via BCA assay under the assumption that any undetected protein was bound to the carrier.

$$Immobilization\ yield = \frac{Enzyme\ added -\ protein\ concentration\ in\ washing\ solution\ (mg)}{Enzyme\ added\ (mg)}$$

(2) Carrier loading

Amount of enzyme bound per weight of carrier.

$$Carrier\ loading\ (mg/g_{carrier}) = \frac{Enzyme\ added\ (mg)}{amount\ of\ immobilization\ carrier\ (g)}$$

(3) Relative catalytic activity

Percentage of specific activity after immobilization relative to that of the free, unbound enzyme.

$$Relative\ catalytic\ activity = \frac{Enzyme\ specific\ activity\ after\ immobilization (U/mg)}{Free\ enzyme\ specific\ activity\ (U/mg)}$$

(4) Activity per carrier

Calculated by multiplying the immobilization yield, carrier loading and enzyme specific activity after immobilization.

$$Activity\ per\ carrier\ (^{U}/_{g_{carrier}}) =$$

$$Immobilization\ yield\ \times Carrier\ loading \times\ Enzyme\ specific\ activty\ after\ immobilization$$

2.4 Characterization of immobilized biocatalysts

The strong affinity between the Strep-Tactin matrix and the Strep-tagged protein accounted for the high immobilization yield of 99% (Table S1). The specific activity for flavin reduction of SH in its free form, initially 2.2 U mg^{-1}, was reduced to 45.5% activity after immobilization. This decrease in activity could be attributed to restricted H_2 diffusion and/or fixed flavins/NAD^+ binding site of the SH, when bound to the Strep-Tactin matrix. Nevertheless, with a protein per carrier loading of 3.3 mg g^{-1} and an FMN reducing activity per carrier of 3.3 U g^{-1} (Table S1), the system demonstrated efficacy in flow chemistry applications.

Different types of EziG beads varying in hydrophilicity were evaluated for *Ts*OYE immobilization to assess performance (see SI chapter 5). All showed similar immobilization yields at 74-78%. The specific activity of free his-tagged *Ts*OYE was 13.5 U mg^{-1}. When immobilized in Amber EziG beads (*Ts*OYE-EziG), it showed a relative catalytic activity of 44.8 %. *Ts*OYE-EziG was immobilized with a higher carrier loading (14.4 mg g^{-1}) than SH-Tactin, resulting in activity per carrier of 66.9 U g^{-1} via NADPH (Table S1). *Ts*OYE-EziG displayed high carrier loading, immobilization yield and relative catalytic activity, comparable to those of OYE3 from *Saccharomyces cerevisiae*, also immobilized on EziG (100% yield, 52% activity) (Tentori et al. 2020). In contrast, *Ts*OYE immobilized on Celite R-648 showed lower support loading and yield, but remarkable stability in high concentration of organic solvents, suggesting potential for improved stability during biocatalytic reduction of alkenes in micro-aqueous organic solvent (Villa et al. 2023).

Table S1. Immobilization of enzymes. Activity measurements were performed in triplicates. Mean and standard deviation are shown

Enzyme	Enzyme carrier	Specific activity free enzyme [U mg^{-1}]	Relative catalytic activity after immobilization [%]	Activity per carrier [U $g^{-1}_{carrier}$]	Immobilization yield [a] [%]	Carrier loading [mg $g^{-1}_{carrier}$]
**SH**	Strep-Tactin XT 4Flow	2.2 ± 0.1 [b]	45.5	3.3	99.0 ± 0.1	3.3
***Ts*OYE**	EziG Bead, Amber	13.5 ± 0.5 [c]	44.8	66.9	77.5 ± 1.3	14.4

[a] Percentage of enzymes bound to the carrier relative to added enzymes. [b] H_2-driven FMN reducing activity. [c] NADPH-driven cyclohexenone reduction activity by his-tagged *Ts*OYE.

3. Continuous flow setup

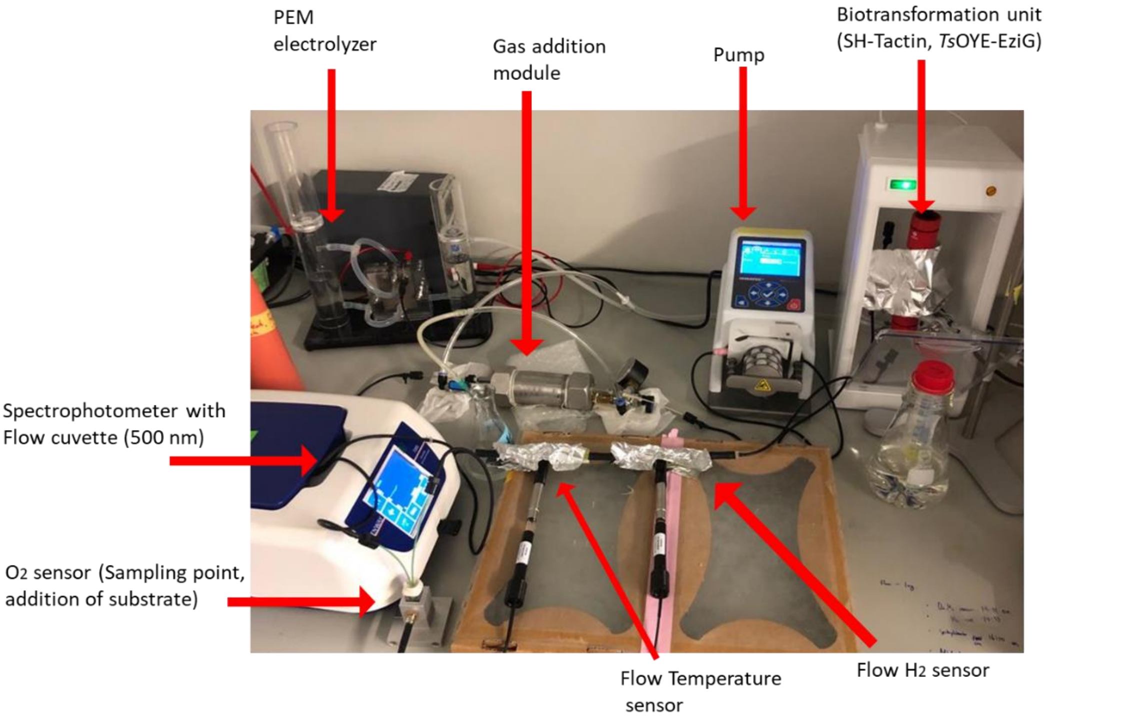

Figure S1. Continuous flow setup with integrated sensors, gas addition module, biotransformation unit, spectrophotometer and PEM electrolyzer.

A gas addition module encased in steel was designed to facilitate the safe transfer of H_2 gas-to-liquid in the flow system (11 cm × Ø 5cm, 150 mL volume). To introduce H_2 to the gas addition module, IQS adapters (Ø 6 mm, R 3/8") equipped with mini ball valve were connected to allow pressurized conditions. Pressure gauge was also equipped measure pressure within the gas addition module. Gas addition module was equipped with a 1/4-28 and 10-32 adapter to be connected to the flow system.
To ensure no contamination of atmospheric gas is permeated through the tubes outside the gas addition module during the reaction, Fluran® F-5500-A tubing with very low gas permeability was used. In addition, the color of the Fluran® tubing was chosen black to block white-light, inhibiting any photo-reduction of FMN in aqueous anaerobic conditions (Song et al. 2007; Mifsud et al. 2014). The amount of dissolved H_2, dissolved O_2 and temperature were measured on-line through an integrated flow sensor to understand the interplay of electrocatalysts and biocatalysts. Modified Clark-type H_2 sensor (UNISENSE) was integrated to the flow setup to measure dissolved H_2. Optical O_2 sensor (PreSens) was integrated with a cuvette attached with an adhesive O_2 spot to measure dissolved O_2. Rubber septa was inserted over the cuvette, to inhibit any atmospheric air while allowing substrate addition. Also, rubber septa allowed addition of substrate or FMN without addition of oxygen. Redox state of cofactor FMN was measured spectrophotometrically at 500 nm wavelength with a flow cuvette on-line during the reaction. Concentration of oxidised FMN state was measured at 500 nm, due to overlapping of absorbance with cyclohexanone and $FMNH_2$ at 320 nm. The temperature of the flow volume was controlled to 30 °C by portable heating cabinet (HARTMANN). The flow rate was constantly set to 2.6 mL min^{-1} to emulate gravity flow rate for SH-Strep, unless stated otherwise. The PEM electrolyzer was set to 0.89 A to produce rate of 11 mL min^{-1} H_2 gas and added to the gas addition module. The outlet of the gas addition module was opened and the outflow was checked through bubbling with a syringe with water.

3.1 Immobilization of biocatalysts, biotransformation unit preparation

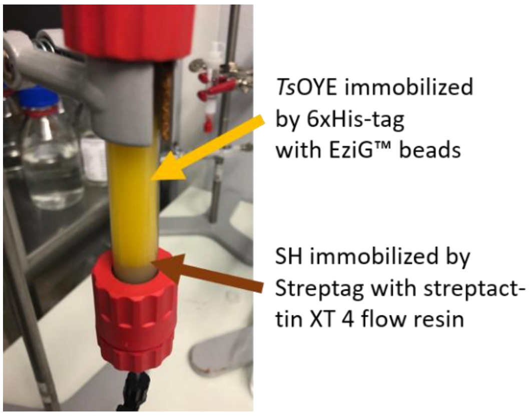

Figure S2. Biotransformation unit packed with biocatalysts and corresponding carriers

- First Pack 1.5 mL of Strep-Tactin XT 4Flow resin into the C 10/10 column. Let it settle overnight in 4 °C.
- Load SH to the Strep-Tactin XT 4Flow (5 mg for 17 mL scale, 6 mg for 185 mL scale)
- Immobilize *Ts*OYE with EziG beads (6.5 mg for 17 mL scale, 8 mg for 185 mL scale). Shake mildly overnight in room temperature.
- Load *Ts*OYE-EziG over the SH-Strep inside the column
- Add catalase inside the column (amount depending on the reaction)
- The biotransformation unit with immobilized biocatalysts are connected to the continuous flow setup

3.2 Electro/-H_2 driven biocatalysis (ketoisophorone as substrate, reusability of the biocatalyst experiments)

- Biotransformation unit is packed with 1.5 mL Strep-Tactin XT 4Flow and loaded with 5 mg of SH, 450 mg of EziG Amber beads were loaded with 6.5 mg of *Ts*OYE as described in 3.1
- Tris-HCl buffer (50 mM, pH 8, 30 °C) is saturated with H_2 by the PEM electrolyzer. Flow rate was set to 2.6 mL min^{-1} using the peristaltic pump.
- FMN stock already saturated with H_2 is added via the septa to make 1 mM concentration to the flow volume (17 mL)
- Observe reduction of FMN to $FMNH_2$ by SH
- When H_2 is re-saturated, substrate ketoisophorone is added into the cuvette via septa along with DMF as a cosolvent, at a ratio of 2:1.

3.3 Electro/-H_2 driven biocatalysis (cyclohexenone as substrate)

The experimental procedure is the same as 3.2 except cyclohexenone is added.

3.4 Electro/-H_2 driven biocatalysis ((*R*)-carvone, (*S*)-carvone as substrate)

The experimental procedure is the same as 3.2 except (*R*)-carvone, (*S*)-carvone are added as substrate. 5 mM concentration is added due to low solubility of carvone to water. The experimental procedure is the same as 3.2 except (*R*)-carvone and (*S*)-carvone are added. The gas permeable tubing is changed to PTFE inside the gas addition module due to observation of adsorption of substrate to PVMS tubing.

3.5 Electro/-H_2 driven biocatalysis (upscale reaction 185 mL, ketoisophorone as substrate)

- Biotransformation unit is packed with 2 mL Strep-Tactin XT 4Flow and loaded with 6 mg of SH 550 mg of EziG Amber beads were loaded with 8 mg of *Ts*OYE as described in 3.1.
- 168 mL volume segment is added with Tris-HCl buffer (50 mM, pH 8, 30 °C).
- The H_2 is saturated in the continuous flow volume with the biotransformation unit. The flow rate of the system was increased to 3.3 mL min^{-1}.
- FMN stock is added via the septa to make up 500 µM FMN concentration in the flow volume
- Observe reduction of FMN to $FMNH_2$ by SH.
- When H_2 is re-saturated, substrate ketoisophorone is added into the cuvette via septa along with DMF as a cosolvent, at a ratio of 2:1.

4. H_2 transfer rate

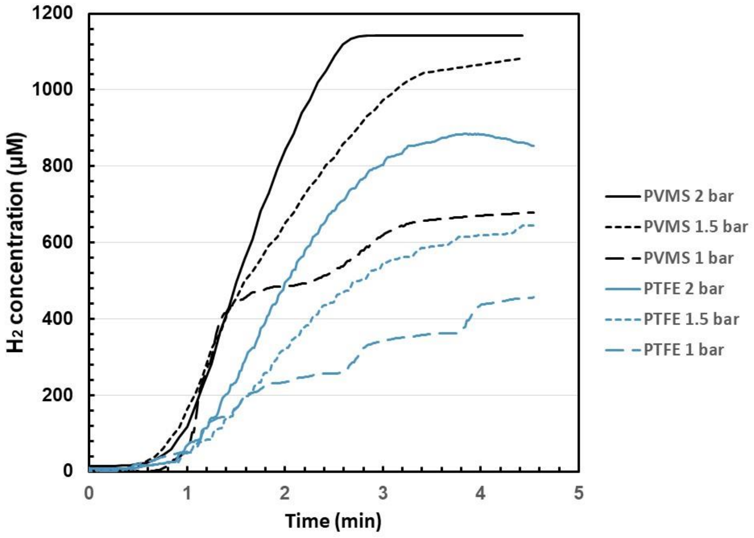

Figure S3. Transfer of H_2 gas to aqueous buffer solutions through a gas-addition module utilizing two different gas-permeable tubing under varying pressure conditions. PVMS tubing (OD = 1.5 mm, ID = 1 mm, length = 2 m), PTFE tubing (OD = 1.5 mm, ID = 1 mm, length = 2 m)

H_2 gas was transferred to the flow volume (50 mM Tris-HCl buffer, pH 8) by introducing it into the enclosed metal casing of the gas addition module, from where it permeated through the 2-meter gas permeable tubing (PVMS or PTFE). To calculate the H_2 transfer from the gas addition module, the dissolved concentration of H_2 was observed. This allowed for the quantification of how much H_2 was transferred from the gas to liquid phase. The experiment was conducted at room temperature (20 °C). The H_2 gas was added to the gas addition module from a pressurized gas cylinder (N5 grade, 99.999% purity). The gas addition module needed to be filled with H_2 to reach maximum gas-to-liquid transfer rate. Therefore, when the dissolved H_2 concentration reached a plateau, which took less than 5 min, this information was used to calculate H_2 transfer rate. For pressurized conditions, the gas output of the gas addition module was closed and the internal pressure was monitored by an integrated pressure gauge.

Under atmospheric pressure, the gas permeable tubes PTFE and PVMS showed gas-to-liquid H_2 transfer reaching a concentration of 440 µM and 680 µM dissolved H_2, respectively after a single pass through the gas addition module (Figure S3). Under elevated pressure at 1.5 bar, dissolved H_2 concentrations reached 640 µM and 1080 µM for PTFE and PVMS, respectively. At 2 bars, the dissolved

H_2 concentrations of 840 µM and 1180 µM were achieved. With the flow rate (2.6 mL min^{-1}) and the volume within the gas permeable tubing (1.57 mL), we were able to calculate the contact time of the buffer during the H_2 transfer (1.65 min). By dividing the contact time from the dissolved H_2 concentration, gas-to-liquid H_2 transfer rate was calculated.

Table S2. H_2 transfer rate *via* gas-permeable tubing inside the gas-addition module.

Gas-permeable tubing (2 m)	Gas-to-liquid transfer rate dissolved H_2 (µmol min^{-1}) *
PTFE	0.418
PVMS	0.647

*H_2 pressure 1 bar, flow rate: 2.6 mL min^{-1}

The difference in H_2 permeability can be ascribed to the variations in their polymeric structures. PVMS has more molecular-level micropores that are created by steric hindrance exerted by the side chains (Özçam et al. 2014). This abundance of micropores leads to higher H_2 diffusion rate in PVMS compared to the more rigid structure of PTFE. Also, during the usage of PVMS or gas permeable tubings, pervaporation of substrate or product with high vapor pressure can be expected (Xiao et al. 2006). Different membranes have varying properties, including whether it is a multilayer structure, that can influence the permeability of gases and volatile chemicals (Baker and Low 2014), expanding the range of tested membrane will give a comprehensive overview for identifying optimal tubing materials.

5. *Ts*OYE immobilization on different EziG beads

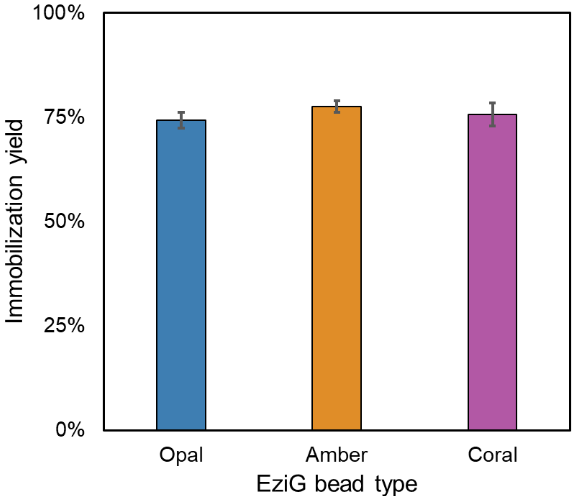

Figure S4. Immobilization yield of *Ts*OYE in different types of EziG beads. Average in triplicates, SD is shown.

Immobilization yield of *Ts*OYE in different EziG beads via coordinate bonds were investigated. EziG Opal has a hydrophilic surface with pure silica surface and no polymer coating. EziG Coral has a hydrophobic surface with poly(vinylbenzylchloride) coating. EziG Amber has a semi-hydrophilic surface with co-polymer (polystyrene derivative). Purified *Ts*OYE was loaded with respective EziG beads with a carrier loading of 14.4 mg $g_{carrier}^{-1}$ and incubated for 30 min with shaking. The immobilization yield was calculated by measuring the concentration of supernatant, assuming the rest of *Ts*OYE were immobilized by the carrier. Amber showed highest immobilization yield by 78% followed by Coral 76% and lastly Opal displaying 74%.

6. Enzymatic membrane reactor (EMR)

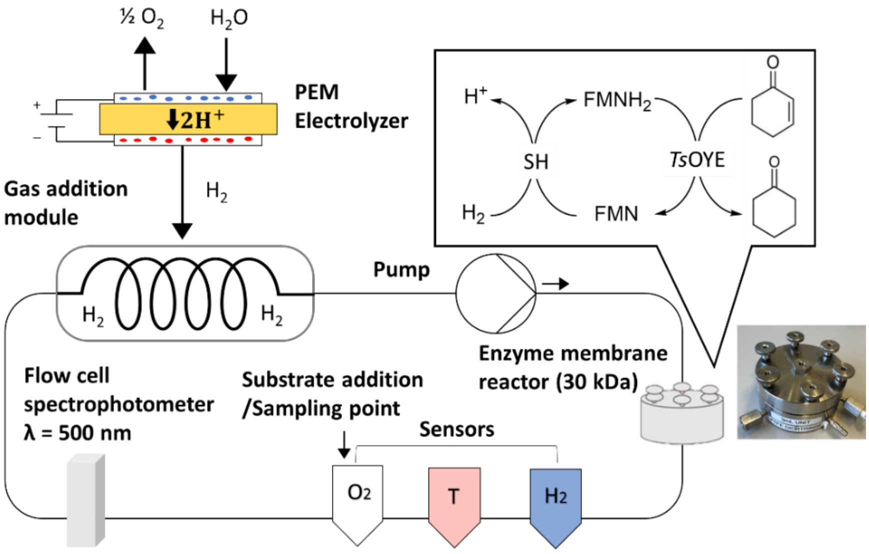

Figure S5. Continuous flow system for electro-driven $FMNH_2$ biocatalysis with integrated enzyme membrane reactor (EMR) for SH, and *Ts*OYE entrapment.

The functionality for the EMR with entrapped SH and *Ts*OYE in the flow reactor was tested. For electro/-H_2 driven biocatalysis in flow setup with EMR, 5 mg of SH and 6.5 mg of *Ts*OYE was added inside the EMR with 30 kDa cellulose membrane (Ultracel®, Merck) on top for entrapment. The rest of the experiment procedure is same as 3.2. The flow volume of the EMR reaction was 25 mL.

7. Electro-driven biotransformation with immobilized enzymes

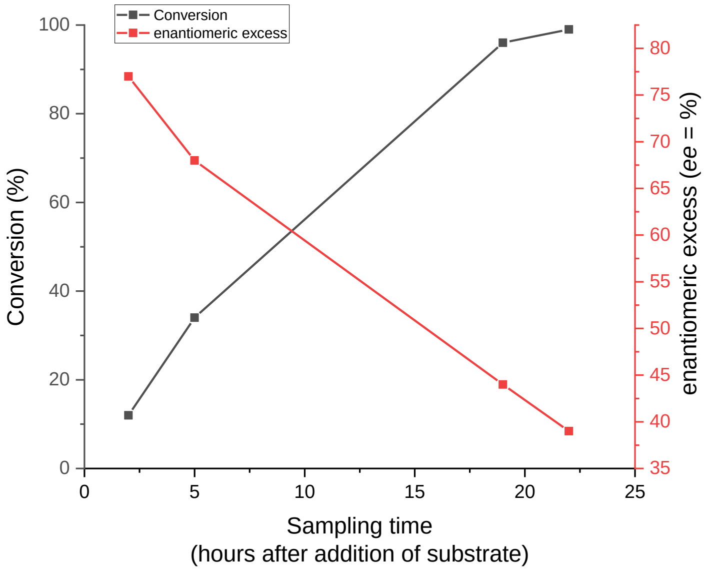

Figure S6. (Top) Conversion (%) and enantiomeric excess (*ee*% *R*) of substrate ketoisophorone to product levodione by time. (Bottom) Conversions of reactions with reused immobilized biocatalysts.

The conversion of ketoisophorone into levodione was tested in the flow system. The electro-driven biotransformation was performed as described in 3.2. Conversion and enantiomeric excess were measured through GC-FID (see 14.2.2).

8. Reusability of the immobilized enzymes

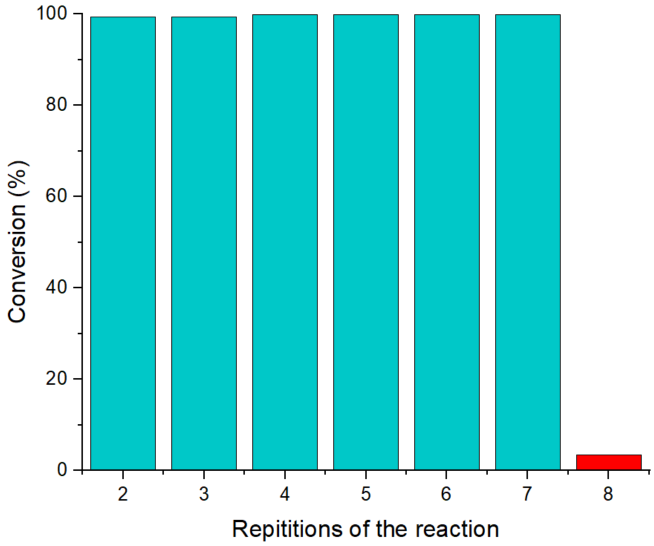

Figure S7. The reusability of the immobilized enzymes in production of levodione from ketoisophorone. Repetitions from 2-7th reactions were conducted in 17 mL conditions (blue). After 7th reaction, the reaction was started in 300 mL volume (red).

The reusability of the immobilized enzymes (1.5 mL Strep-Tactin XT 4Flow loaded with 5 mg of SH, 450 mg of EziG Amber beads loaded with 6.5 mg of *Ts*OYE) in the flow reactor was tested. The conversion of ketoisophorone to levodione was analysed by GC-FID after multiple runs. Here, the reaction was performed as described in 3.2. Each reaction was performed overnight and the samples were taken 19 h after addition of substrate ketoisophorone. After the reaction was finished, the biotransformation unit was removed from the setup, and the flow system was washed out and equilibrated with a new buffer and a new reaction was started. In the 8th repetition, 300 mL of volume in a Schott bottle was attached to the flow setup to test bigger scaled reaction. The reaction was stopped at 4% due to presence of O_2 in the headspace of the Schott bottle, with the enzymes being inactivated by H_2O_2 generated by $FMNH_2$.

9. Total turnover number for reused immobilized biocatalysts

Table S3. Total turnover numbers (TTN $n_{product}/n_{enzyme}$)of each biocatalyst based on the sum of products formed after the using the same set of immobilized enzymes.

Reaction	SH-TTN	*Ts*OYE-TTN	Reference
After 7th reuse in flow system (17 mL)	1.01×10^5	1.69×10^4	This work
After 2nd reuse in flow system (185 mL)	3.21×10^5	2.65×10^4	This work
Biphasic reaction	n.a.	Over 1.75×10^4	(Nett et al. 2021)

10. Conversion rate during upscaled reaction

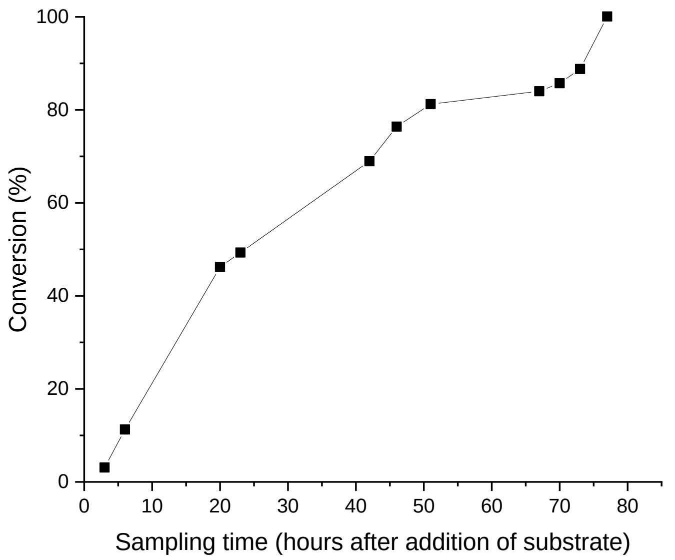

Figure S8. Conversion rate across timepoints during the upscaled reaction (185 mL) of electro/-H_2 driven asymmetric reduction of ketoisophorone to levodione by *Ts*OYE

The conversion of ketoisophorone to levodione was tested in the upscaled flow system. Here, the reaction was performed as described in 3.5. The reaction reached full conversion after 77 h (Figure S8).

11. Comparison with literature

Table S4. List of parameters and conversions with *Ts*OYE from previous studies in comparison with the current work. Cofactor was compared with other literatures.

Substrate	Volume (mL)	Concentration (mM)	Cofactor	Yield (%)	Cofactor -TNN*	Reference
**Cyclohexenone**	1	10	NADPH	99.9	100	(Jongkind et al. 2022)
	17	25	$FMNH_2$	35.0	8.75	This Work
	2	22	$FMNH_2$	44.2	48	(Al-Shameri et al. 2020)
**Ketoisophorone**	0.050	10	$FMNH_2$	20.0	2.35	(Son et al. 2018)
	1.5	10	$FMNH_2$	60.0	30	(Gonçalves et al. 2019)
	0.6	24.2	$FMNH_2$	>99	240	(Joseph Srinivasan et al. 2021)
	17	25	$FMNH_2$	99.9	25	This Work
	185	18.5	$FMNH_2$	99.9	37	This Work
	2	22	$FMNH_2$	99,9	105	(Al-Shameri et al. 2020)
**(*R*)-(–)-Carvone**	17	5	$FMNH_2$	14.0	5	This work
	2	22	$FMNH_2$	5.0	5.5	(Al-Shameri et al. 2020)
	18	5	$FMNH_2$	1.3	0.65	(Tosstorff et al. 2017)
	18	5	NADPH	1.8	0.92	(Tosstorff et al. 2017)
**(*S*)-(+)-Carvone**	17	5	$FMNH_2$	34.0	5	This Work
	2	22	$FMNH_2$	8.6	9.5	(Al-Shameri et al. 2020)

*Total turnover number TTN ($n_{product}/n_{cofactor}$) of mol product per mol cofactor

12. Faradaic efficiency

(1) $$Faradaic\ efficiency\ (\%) = \frac{Actual\ electrons\ used}{Total\ electrons\ passed} = \frac{\alpha n F}{Q} \times 100$$

$\alpha =$ amount of levodione reduced (mol)
$n =$ electrons needed (n = 2)
$F =$ Faraday constant (96485 C mol^{-1})
$Q =$ total charges passed during the reaction as H_2 gas (current (A) x time (s))

Faradaic efficiency of the continuous flow system coupled with a commercial PEM electrolyzer was determined with an electro-driven reduction reaction from **3** to **4**. The preparation of the experiment is the same as chapter 3.1 except the flow volume (17 mL) of the system was purged of O_2 with N_2 gas through the gas addition module. Then, FMN and substrate **3** were added to achieve concentrations of 1 mM and 25 mM, respectively. Before turning on the PEM electrolyzer, the N_2 in the gas addition module was quickly flushed out with H_2 gas from a gas cylinder. This procedure was aimed to minimize the time of electrolysis during which the gas N_2 gas within the gas addition module volume (150 mL) transitions to H_2. PEM electrolyzer (H-Tec education) was set to 3.3 V, 0.5 A (H_2 0.7 mL min^{-1}), which was the lowest potential and current to start stable electrolysis of water. The reaction was stopped after 24 hours where 85 % conversion was observed. The Faradaic efficiency was calculated according to the Equation 1. H_2 gas that was used to flush the gas addition module volume (150 mL, 1 atm, 20 °C) was also accounted as total electrons passed. The Faradaic efficiency of the continuous flow system for 3 to 4 reaction was calculated to be 0.15 %.

13. Environmental impact

(2) $$E = \frac{\sum m_{waste}}{m_{product}} \left[\frac{kg}{kg}\right]$$

E factors were roughly calculated to estimate the environmental impact of the reaction (Equation. 2) (Sheldon 2017). Ketoisophorone to levodione in 17 mL and upscaled (185 mL) reaction were compared.

Table S5. *E* factor comparison between ketoisophorone reactions in flow system with different volumes

**Entry**	**Reaction, volume**	**Mass$_{waste}$ components**[a]	**Waste (mg)**[b]	**Mass$_{product}$ (mg)** [c]	***E* factor**
1	H_2-driven *Ts*OYE reaction by SH in 17 mL volume (used 7 times)	Tris (50 mM)	102.8 × 7	65.5 × 7	6.0
		FMN (1 mM)	7.7 × 7		
		Isolated SH	5		
		Isolated *Ts*OYE	6.5		
		Strep-Tactin XT 4Flow	1500		
		EziG beads	450		
		Catalase	5 x 7		
2	H_2-driven *Ts*OYE reaction by SH in 185 mL volume (used 2 times)	Tris (50 mM)	1119.3 × 2	527.8 + 364.2	5.7
		FMN (500 µM)	42.2 × 2		
		Isolated SH	6		
		Isolated *Ts*OYE	8		
		Strep-Tactin XT 4Flow	2000		
		EziG beads	550		
		Catalase	25 x 2		
		Unreacted ketoisophorone	161.5		

[a] Waste component were determined using Tris-HCl buffer at pH 8 (with indicated concentration). No side reactions were observed during this reaction. In entry 2, ketoisophorone mass was calculated based on 69% conversion (based on GC-FID result, 13.1.2). Mass from cultivation, purification step and H_2 gas were not included as waste components.
[b] The amount of waste from buffer and FMN was measured as the reaction were repeated for the immobilized enzymes.
[c] The production of levodione as a product was quantified as the reaction was repeated. For entry 2, 69% product formation was accounted. Theoretical yield was counted for Mass$_{product}$ calculations.

14. GC analyses

14.1 GC-FID

Table S6. GC-FID parameters

Column	Temperature gradient	Analyte (retention time, min)
J&W VF-5ms GC Column (Agilent),<br>Part number CP9013<br>(30 m × 0.25 mm × 0.24 µm)<br>Carrier gas: He<br>Split ratio: 10<br>Injection volume: 1 µL	80 °C hold for 3 min<br>25 °C min^{-1} to 300 °C hold 2 min	Cyclohexenone (5.95)<br>Cyclohexanone (5.55)<br>Acetophenone (7.24)<br>Ketoisophorone (7.8)<br>Dodecane (7.95)<br>Levodione (8)<br>(*R*)-Carvone, (*S*)-carvone (8.5)<br>(1*S*, 4*S*)-Dihydrocarvone,<br>(1*R*, 4*R*)-Dihydrocarvone (8.19)<br>(1*R*, 4*S*)-Dihydrocarvone,<br>(1*S*, 4*R*)-Dihydrocarvone (8.23)

14.1.1 Ketoisophorone to levodione

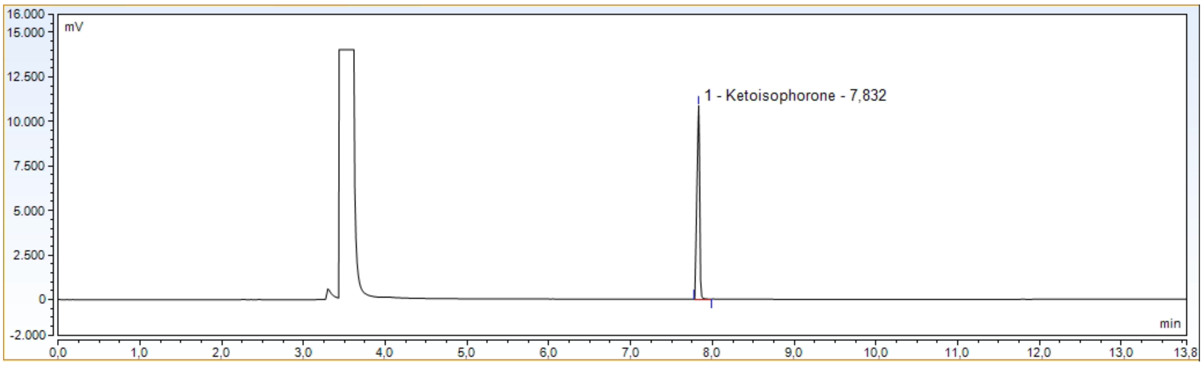

Figure S9-A. Ketoisophorone standard

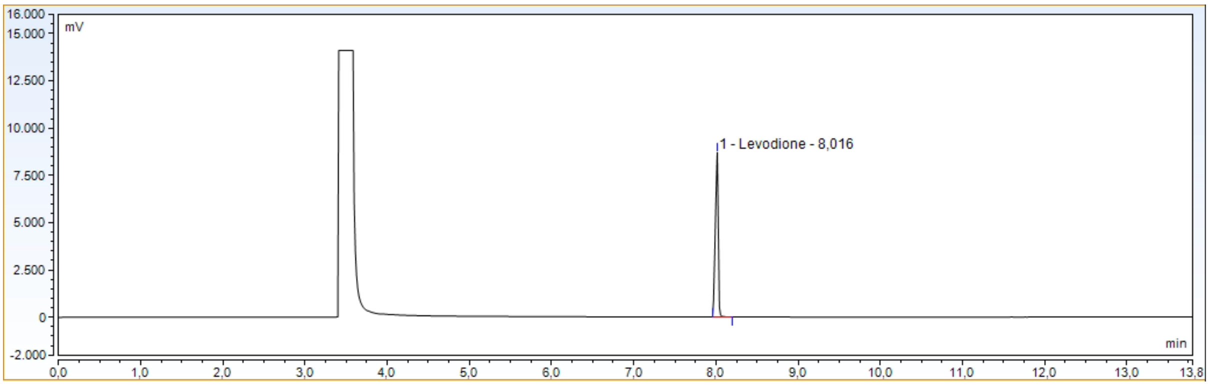

Figure S9-B. (6*R*)-Levodione standard

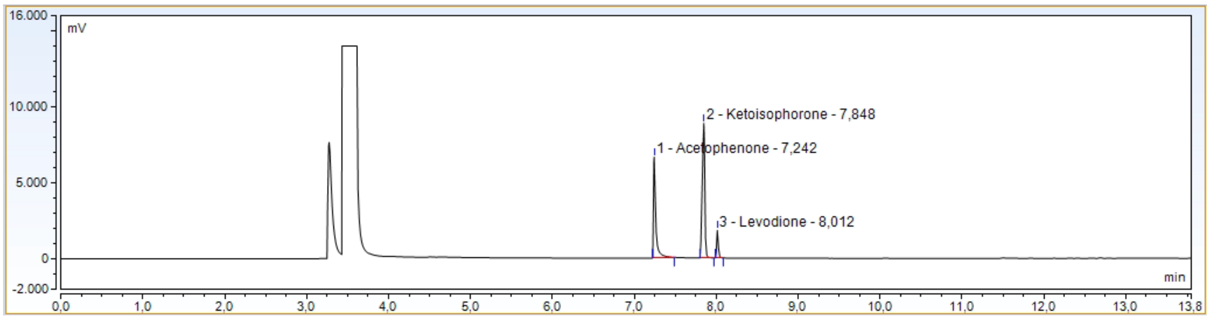

Figure S9-C. 2 h after addition of substrate (17 mL reaction).

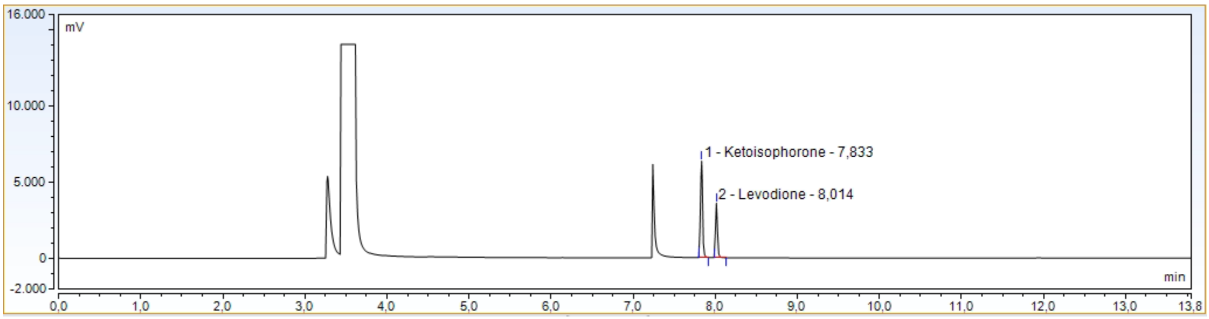

Figure S9-D. 5 h after addition of substrate (17 mL reaction). Internal standard acetophenone peak at 7.24 min.

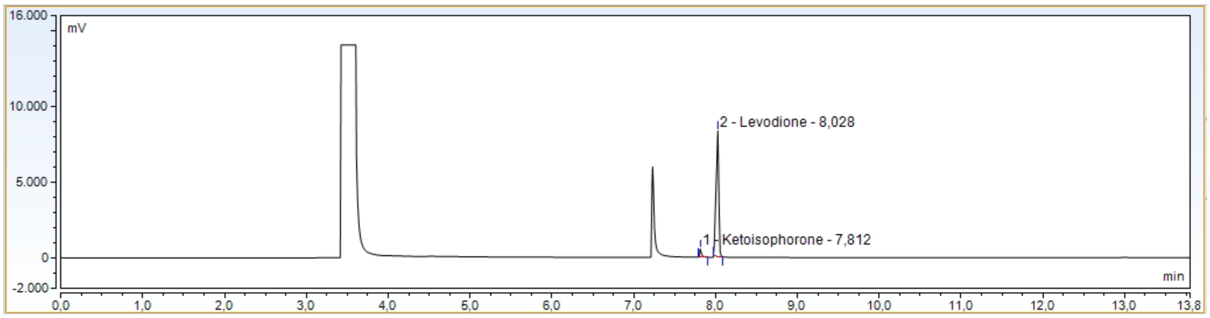

Figure S9-E.19 h after addition of substrate (17 mL reaction). Internal standard acetophenone peak at 7.24 min.

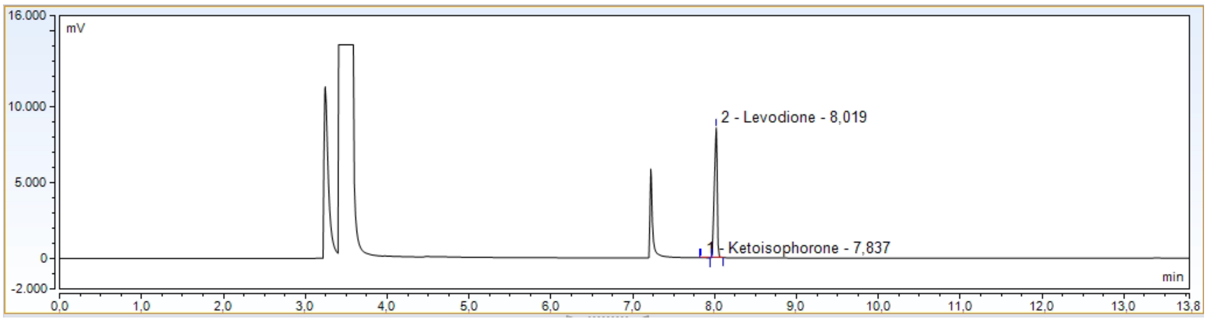

Figure S9-F. 22 h after addition of substrate (17 mL reaction). Internal standard acetophenone peak at 7.24 min.

14.1.2 Ketoisophorone to levodione (Upscaled reaction)

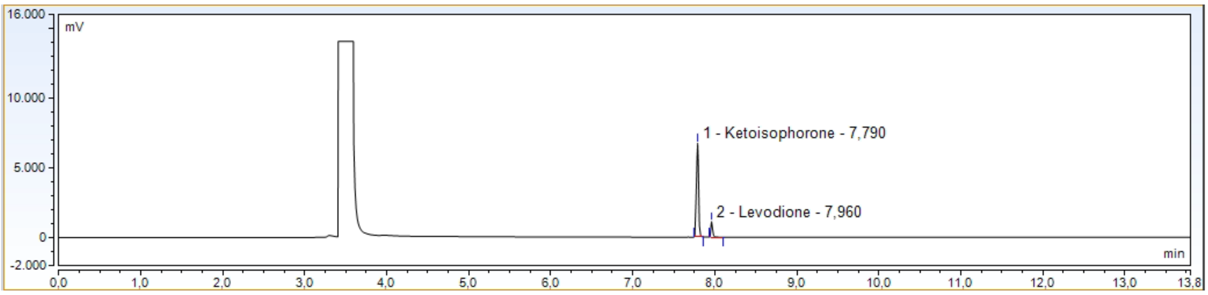

Figure S10-A. 3 h after addition of substrate (185 mL reaction)

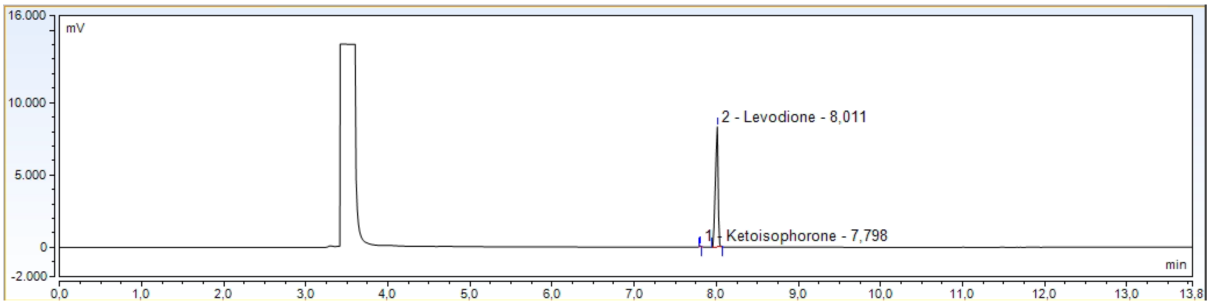

Figure S10-B. 77 h after addition of substrate (185 mL reaction)

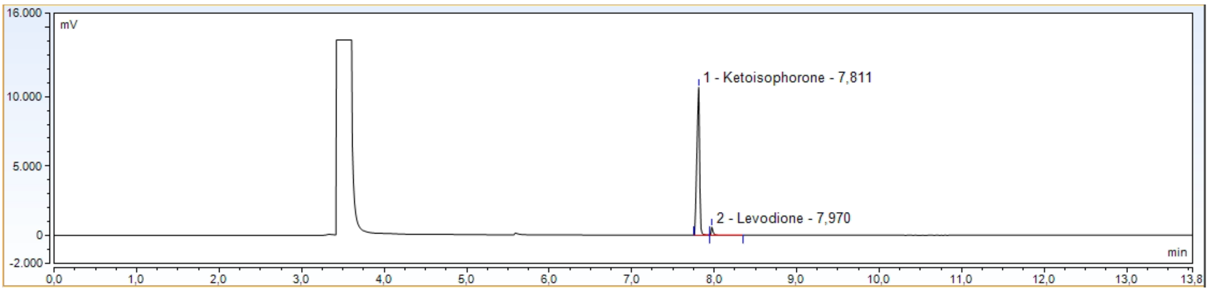

Figure S10-C. 1 hour after addition of substrate, 2nd use of immobilized enzymes (185 mL reaction)

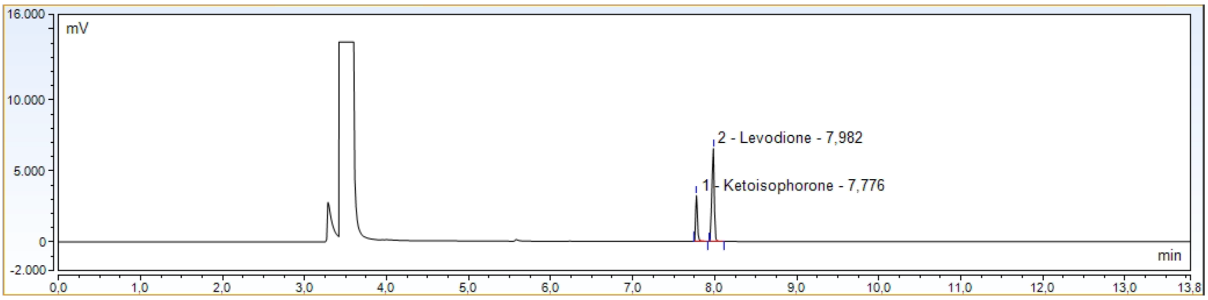

Figure S10-D. 46 h after addition of substrate, 2nd use of immobilized enzymes (185 mL reaction)

14.1.3 Cyclohexenone to cyclohexanone

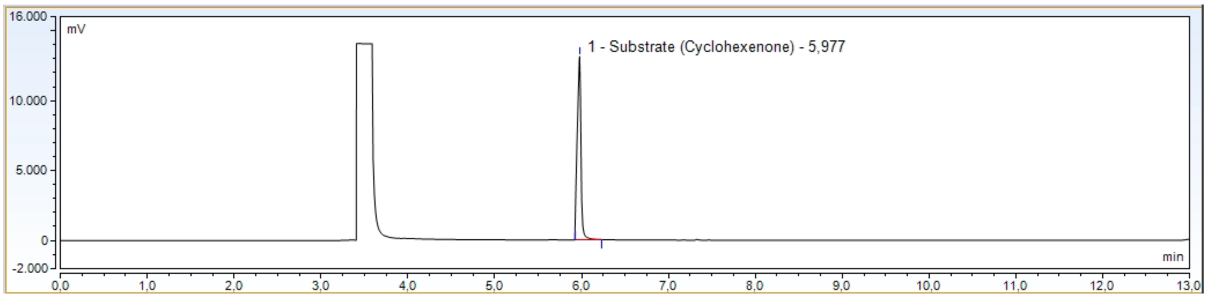

Figure S11-A. Cyclohexenone standard

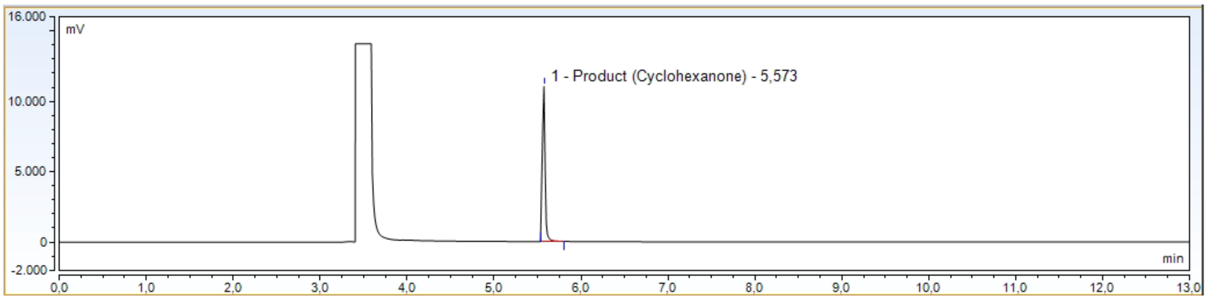

Figure S11-B. Cyclohexanone standard

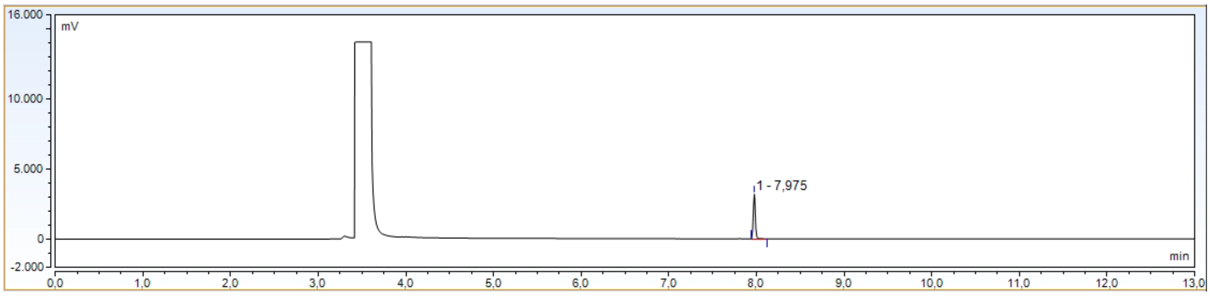

Figure S11-C. Negative sample (17 mL reaction). Internal standard dodecane peak at 7.975 min.

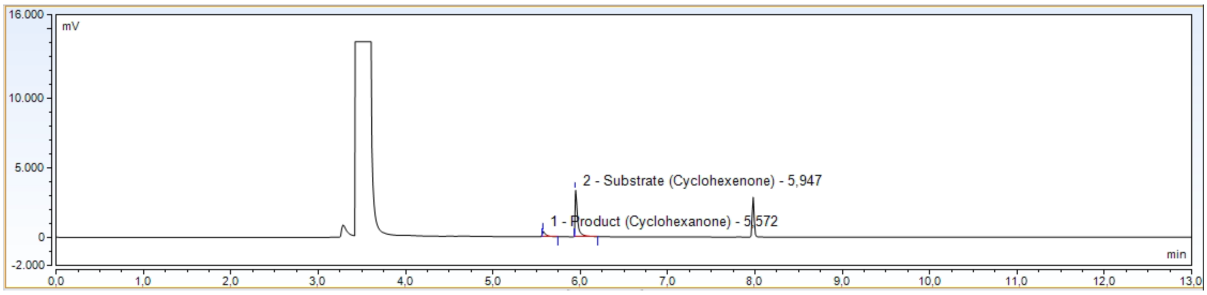

Figure S11-D. 1 hour after addition of substrate (17 mL reaction). Internal standard dodecane peak at 7.975 min.

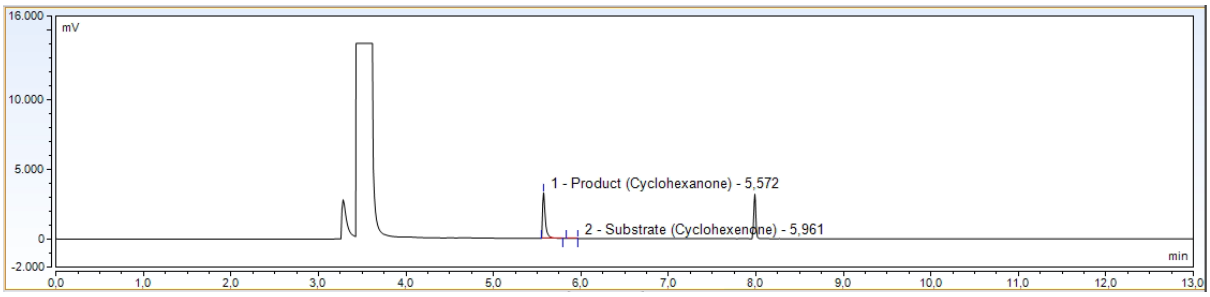

Figure S11-E. 16 h after addition of substrate (17 mL reaction). Internal standard dodecane peak at 7.975 min.

14.1.4 Cyclohexenone to cyclohexanone with EMR

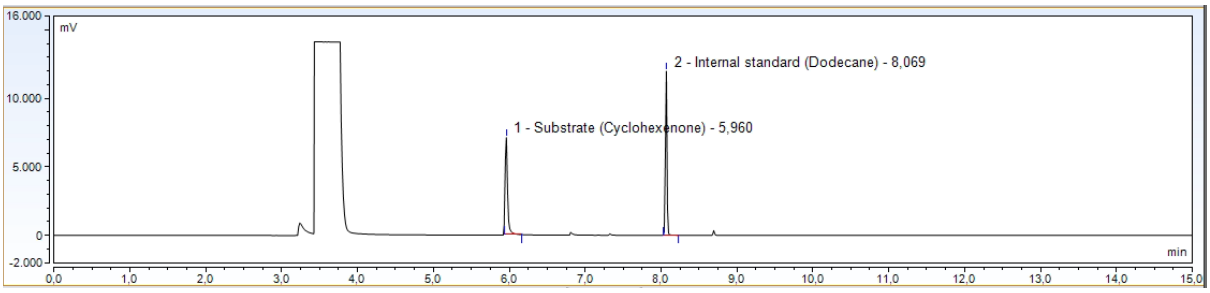

Figure S12-A. 30 min after addition of substrate (EMR, 25 mL reaction).

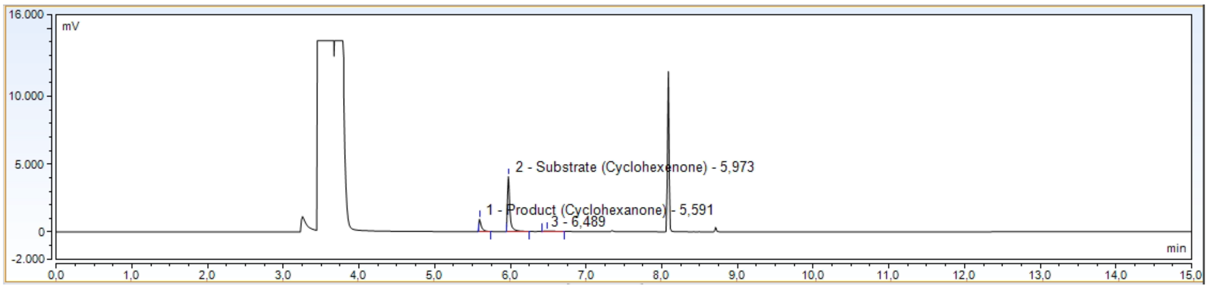

Figure S12-B. 2 days after addition of substrate (EMR, 25 mL reaction). Internal standard dodecane peak at 8.069 min. Side product observed at 6.489 min.

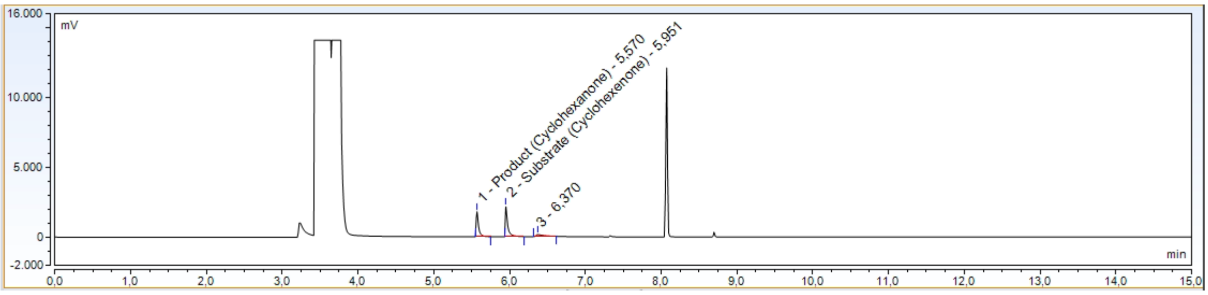

Figure S12-C. 6 days after addition of substrate (EMR, 25 mL reaction). Internal standard dodecane peak at 8.069 min. Side product observed at 6.489 min.

14.1.5 (*R*)-(-)-Carvone to (+)-Dihydrocarvone

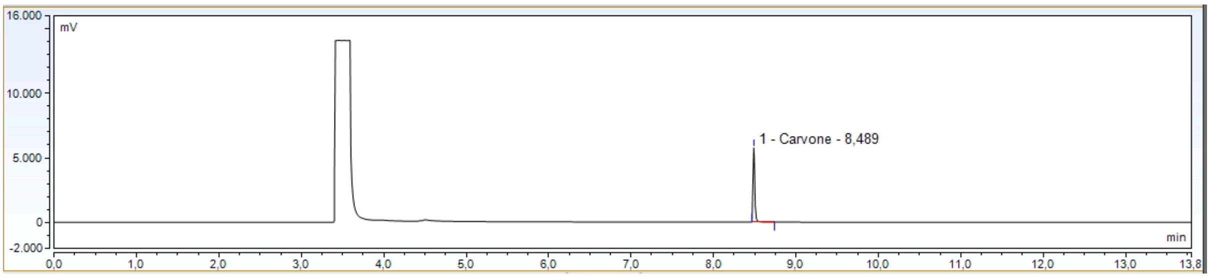

Figure S13-A. (*R*)-Carvone standard

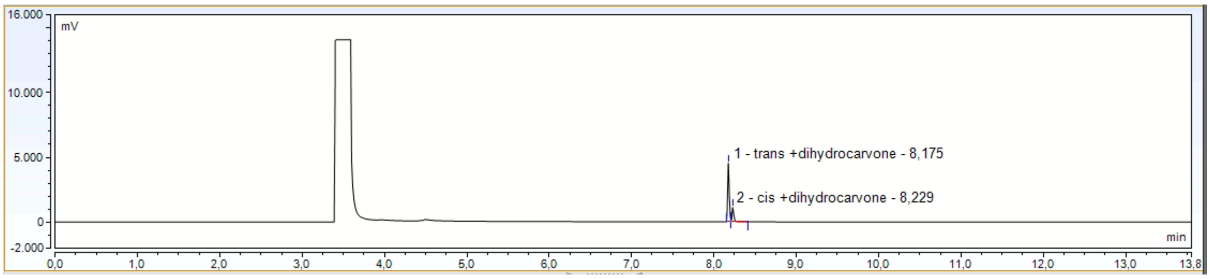

Figure S13-B. (+)-Dihydrocarvone standard (n-(+)-dihydrocarvone 77 %, iso-(+)-dihydrocarvone 20 %)

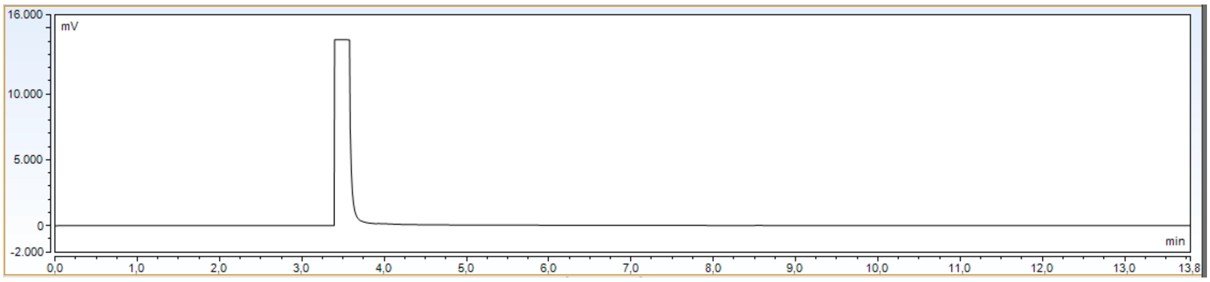

Figure S13-C. Negative sample (17 mL reaction).

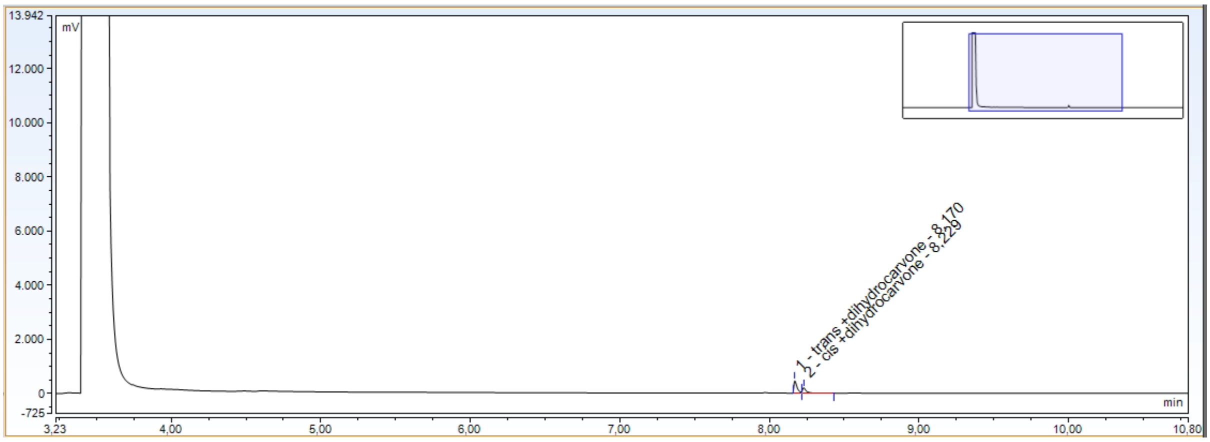

Figure S13-D. 16 h after substrate (*R*)-Carvone addition (17 mL reaction).

14.1.6 (*S*)-(+)-Carvone to (-)-Dihydrocarvone

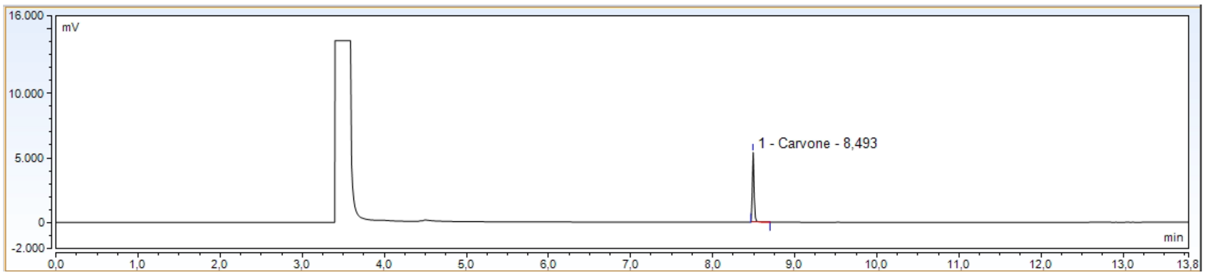

Figure S14-A. (S)-Carvone standard

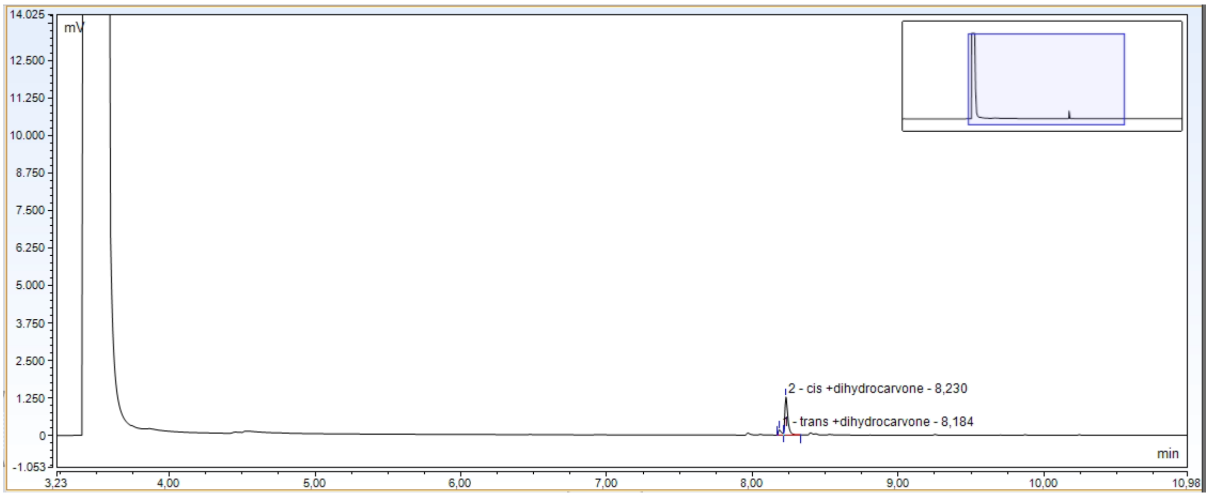

Figure S14-B. 16 h after addition of substrate (*S*)-carvone (17 mL reaction).

14.2 GC-FID Chiral

GC: Shimadzu GC-2010 gas chromatographs (Shimadzu corporation, Kyoto, Japan) equipped with a flame ionization detector (FID)

Table S7. Chiral GC-FID parameters

Column	Temperature gradient	Analyte (retention time, min)
Macherey-Nagel Lipodex™ E<br>(50 m × 0.25 mm × 0.25 µm)<br>Carrier gas: He<br>Split ratio: 100<br>Injection volume: 1 µL<br>Injection temp: 250 °C<br>Detector temp: 275 °C<br>linear velocity 38 cm/s	80 °C hold for 2 min<br>5 °C min^{-1} to 110 °C hold 5 min<br>5 °C min^{-1} to 130 °C hold 5 min<br>20 °C min^{-1} to 220 °C hold 1 min	(*R*)-Carvone (19.55)<br>(*S*)-Carvone (19.45)<br>(1*S*, 4*S*)-Dihydrocarvone (16.3)<br>(1*R*, 4*R*)-Dihydrocarvone (16.5)<br>(1*R*, 4*S*)-Dihydrocarvone (17.4)<br>(1*S*, 4*R*)-Dihydrocarvone (17.9)
Macherey-nagel Hydroxdex β-TBDAc (50m x0.25 m x0.25 µm)<br>Carrier gas: He<br>Split ratio: 50<br>Injection volume: 1 µL<br>Injection temp: 250 °C<br>Detector temp: 250 °C<br>linear velocity 38 cm/s	100 °C hold for 3.5 min<br>15 °C min^{-1} to 250 °C hold 1 min	Ketoisophorone (10.3)<br>(*R*)-Levodione (10.5)<br>(*S*)-Levodione (10.7)

14.2.1 Carvone as substrate

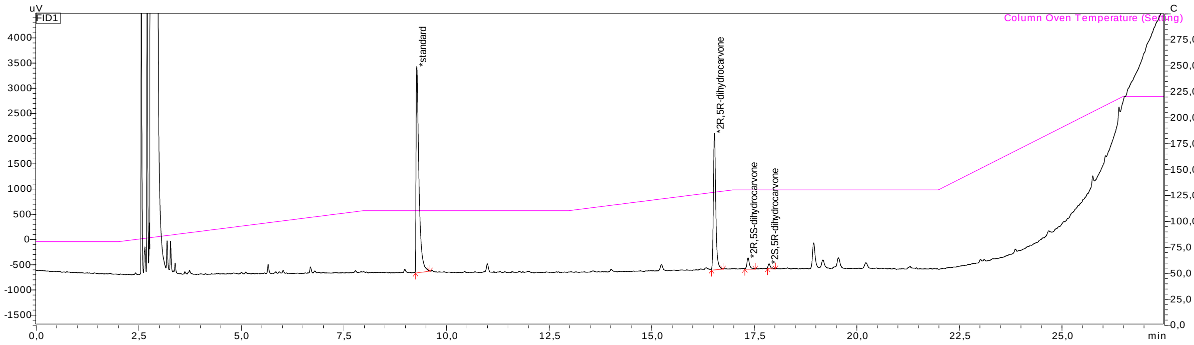

Figure S15-A. (-)-Carvone to (+)-dihydrocarvone sample detected by chiral column

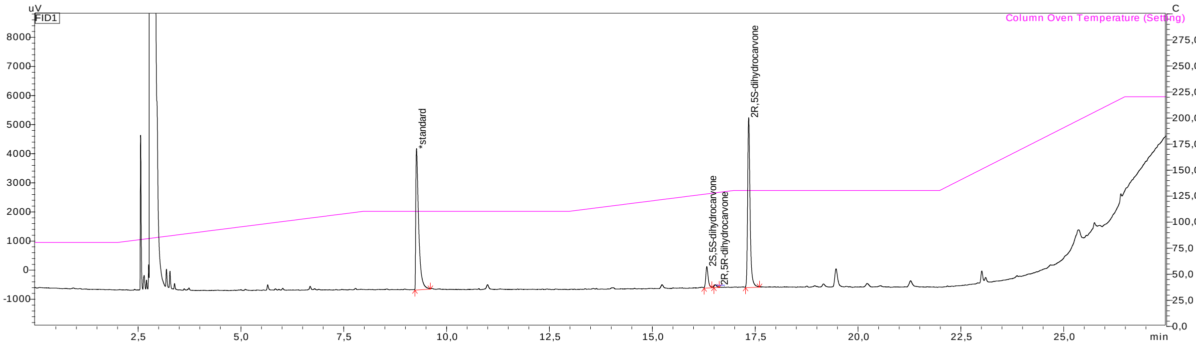

Figure S15-B. (+)-Carvone to (-)-dihydrocarvone sample detected by chiral column

14.2.2 Ketoisophorone as substrate (Reusability experiment)

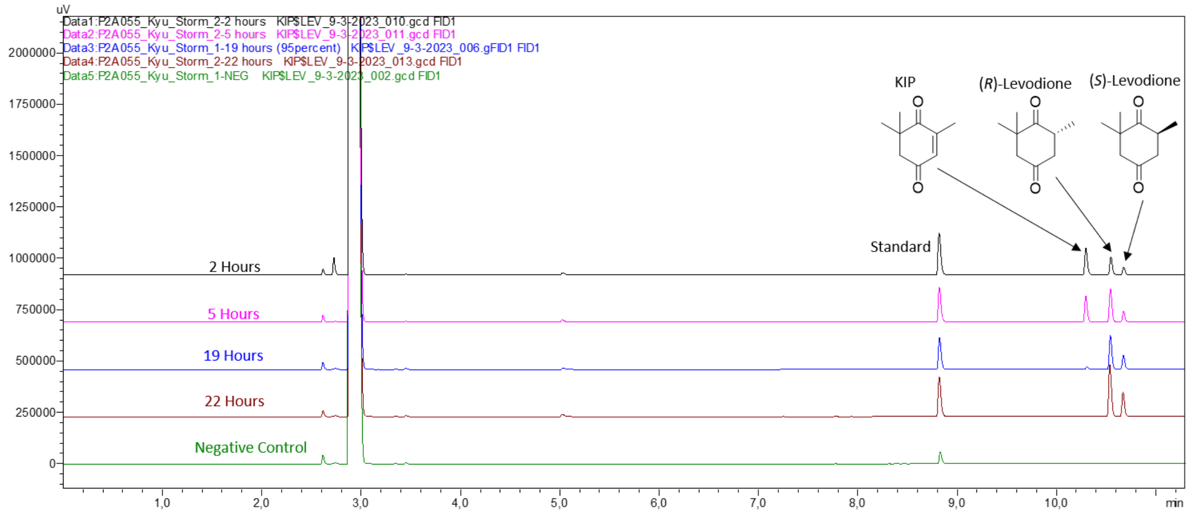

Figure S16. GC-FID chromatogram comparison of biotransformations of KIP (Ketoisophorone or 2,6,6-trimethylcyclohex-2-ene-1,4-dione) towards Levodione (2,2,6-trimethylcyclohexane-1,4-dione) with biocatalyst *Ts*OYE. Shown are data peaks from 2 h (12% conversion and *ee* 77%(*R*)) , 5 h (34% conversion and *ee* 68%(*R*)), 19 h (96% conversion and *ee* 44%(*R*)), 22 h (100% conversion and *ee* 39%(*R*)) and a negative control. Peak at 5 min is DMF.

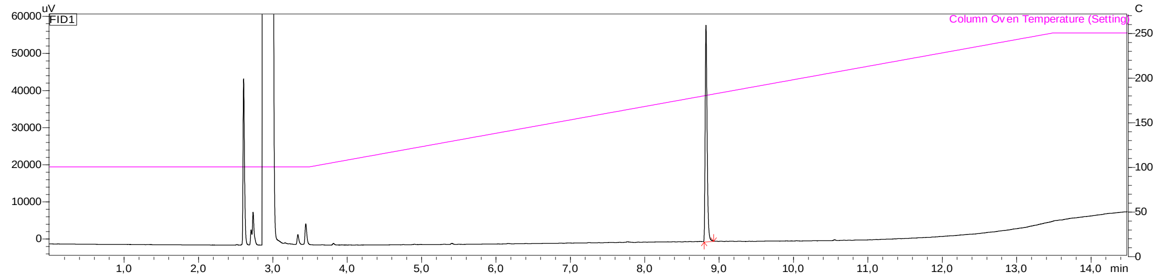

Figure S176-A. Negative control

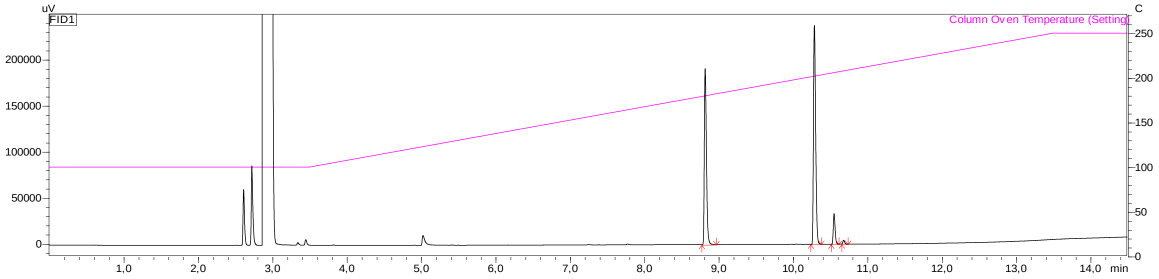

Figure S16-B. 2 h after addition of substrate (12% conversion, *ee* = 77 % *R*)

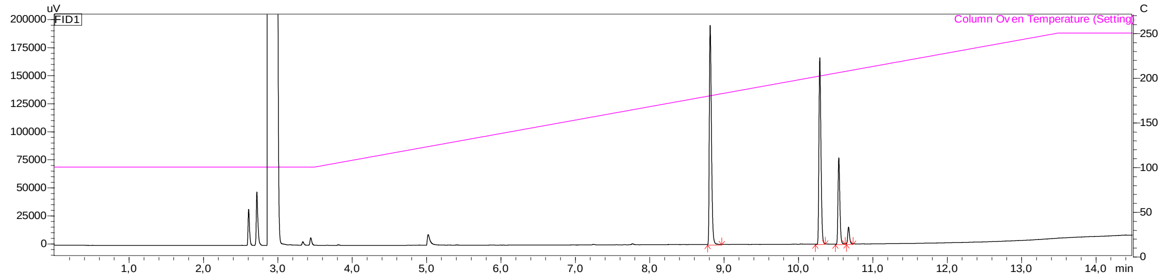

Figure S16-C. 5 h after addition of substrate (34% conversion, *ee* = 68% *R*)

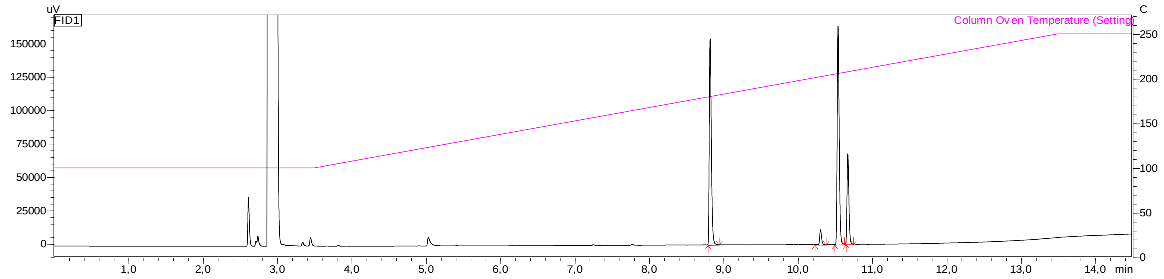

Figure S16-D. 19 h after addition of substrate (95.7% conversion, *ee* = 44% *R*)

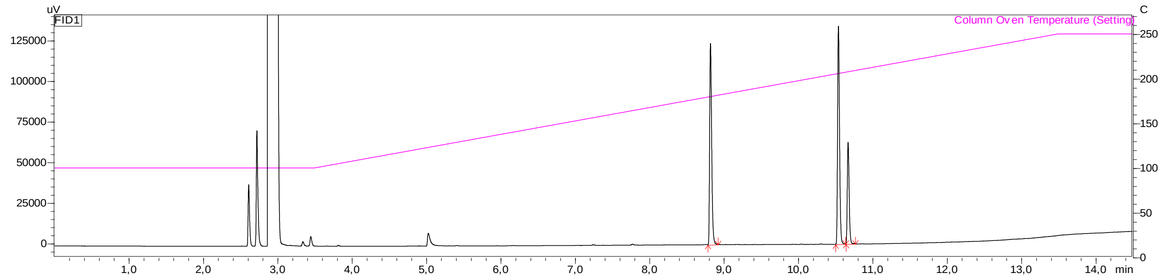

Figure S16-E. 22 h after addition of substrate (100% conversion, *ee* = 39% *R*)

14.3 GC-MS

Table S8. GC-MS parameters

Column	Temperature gradient	Analyte (retention time, min)
Agilent VF-5ms column<br>(30 m × 0.25 mm × 0.25 µm)<br>Carrier gas: H_2 (30 cm s^{-1})<br>Split ratio: 10<br>Injection volume: 1µL	Start at 50°C<br>Ramp to 200°C at 10°C/min<br>Hold at 200°C for 5 mins<br>Ramp to 300°C at 10°C/min<br>Hold at 300°C for 5 mins	Cyclohexanone (3.86)<br>Hydroxylated side-product **1** (6.18)<br>Hydroxylated side-product **2** (3.1)<br>Hydroxylated side-product **3** (3.8)

14.3.1 Cyclohexanone

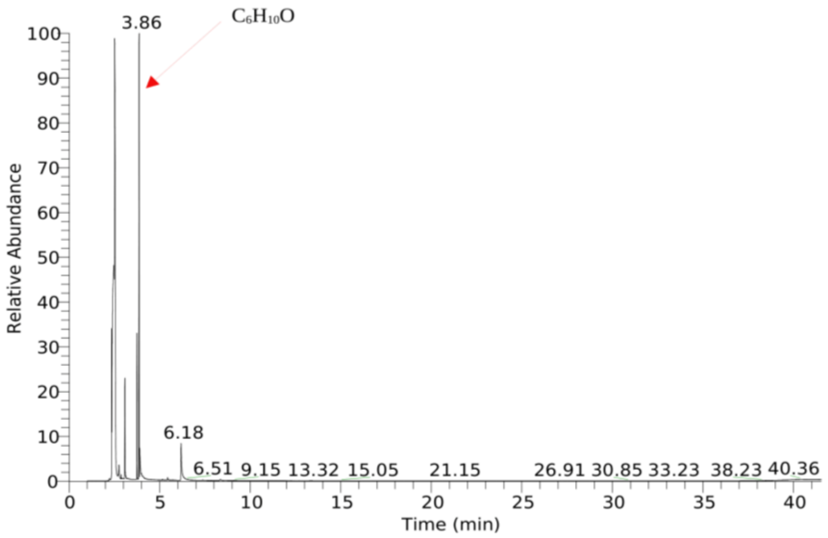

Figure S17-A. GC Chromatogram of cyclohexanone

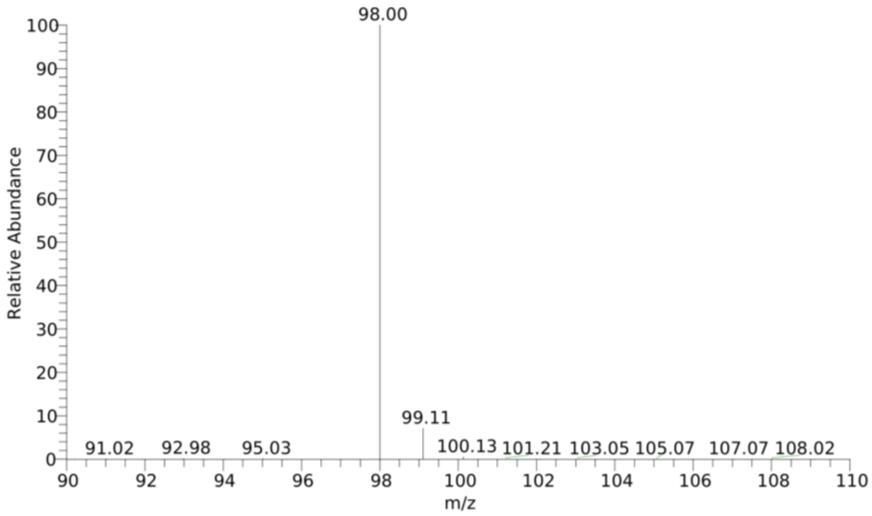

Figure S17-B. MS spectra of the peak

14.3.2 Hydroxylated side product **1** (Top: GC chromatogram, bottom: MS spectra of the respective peak)

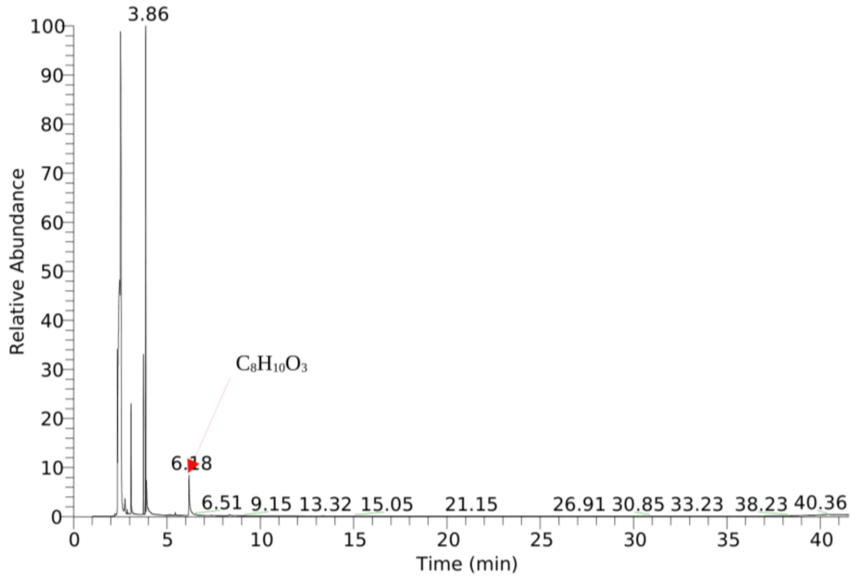

Figure S18-A. GC chromatogram of hydroxylated side product **1**

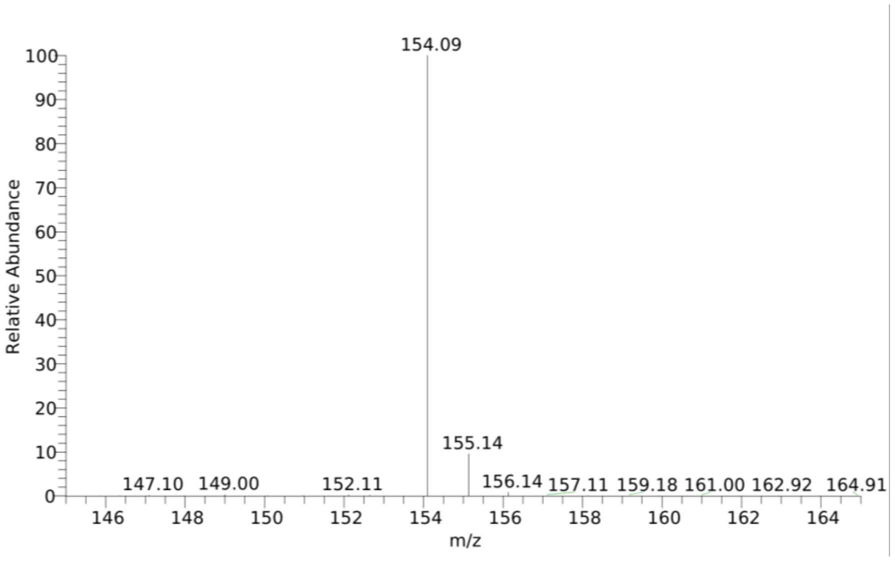

Figure S18-B. MS spectra of the respective peak

14.3.3 Hydroxylated side product **2**

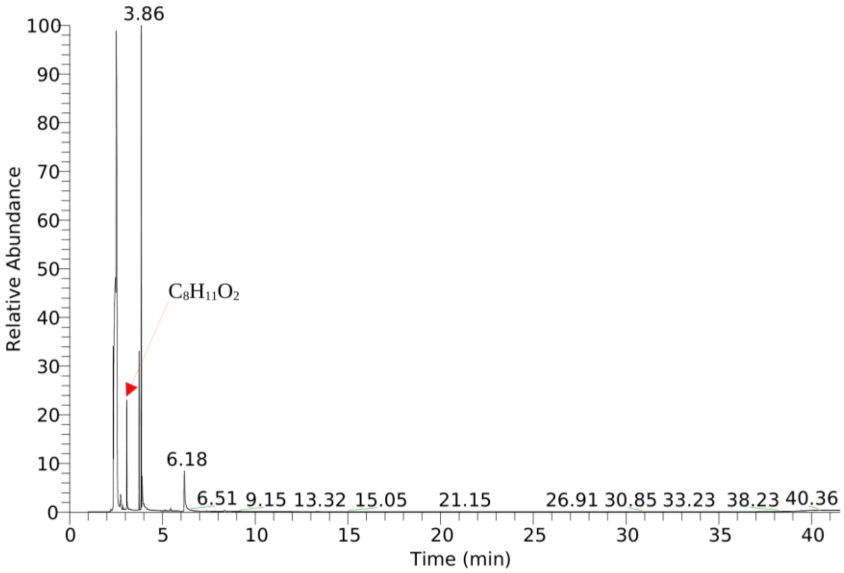

Figure S19-A. GC chromatogram of hydroxylated side product **2**

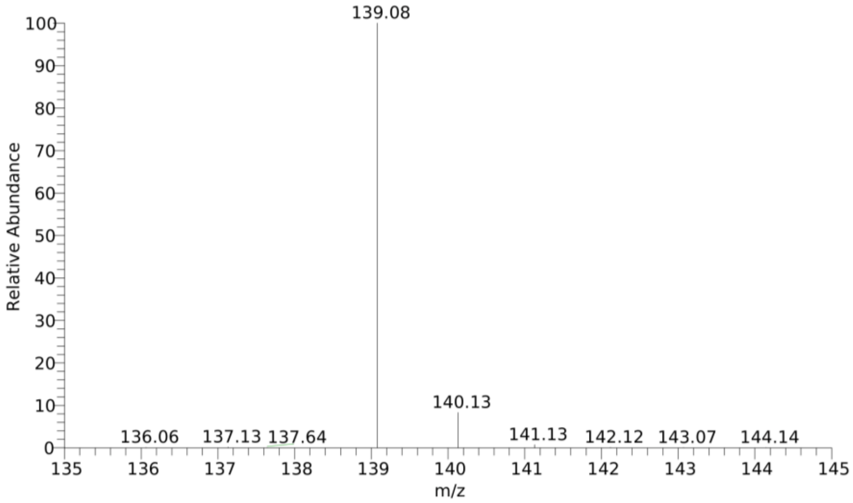

Figure S19-B. MS spectra of the respective peak

14.3.4 Hydroxylated side product **3**

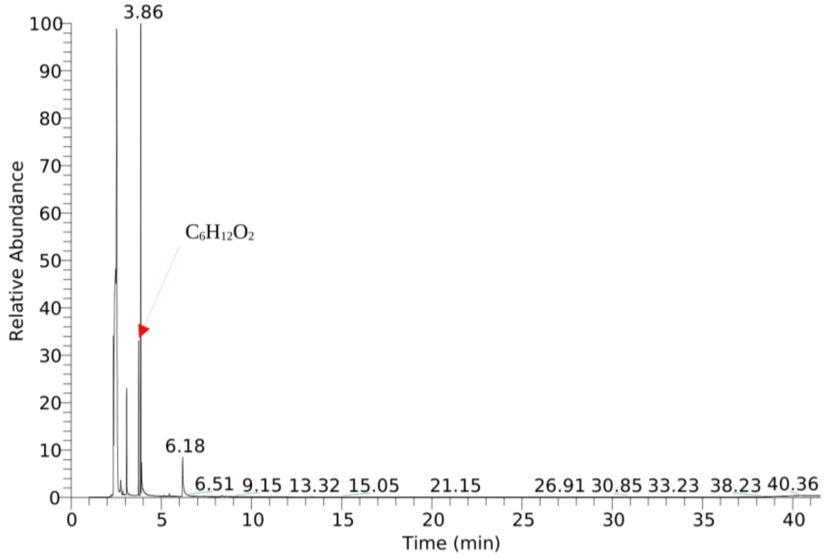

Figure S20-A. GC chromatogram of hydroxylated side product **3**

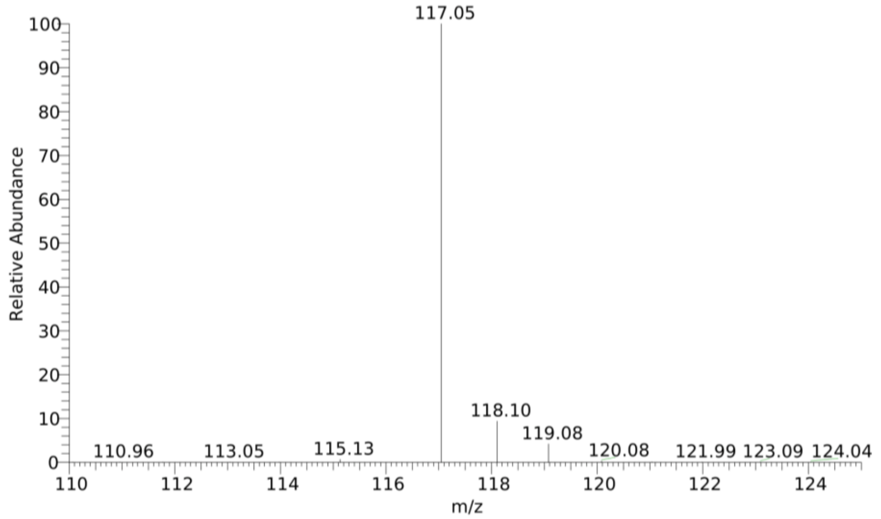

Figure S20-B. MS spectra of the respective peak

15. ^{1}H Nuclear magnetic resonance spectra of the isolated levodione

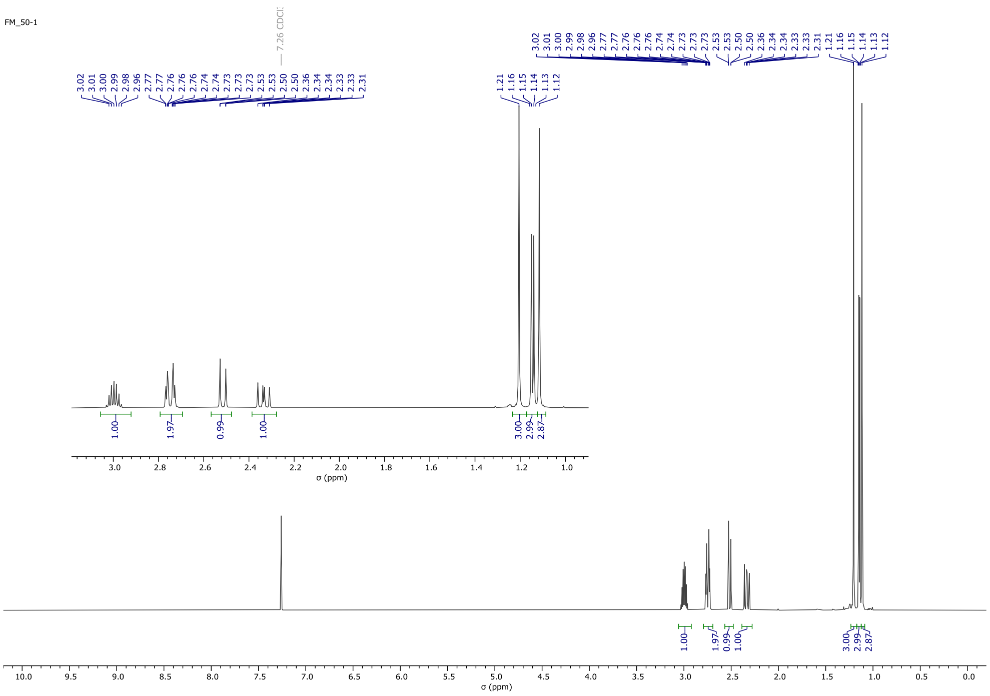

Figure S21. ^{1}H NMR spectrum ($CDCl_3$, 600 MHz) of the isolated levodione.

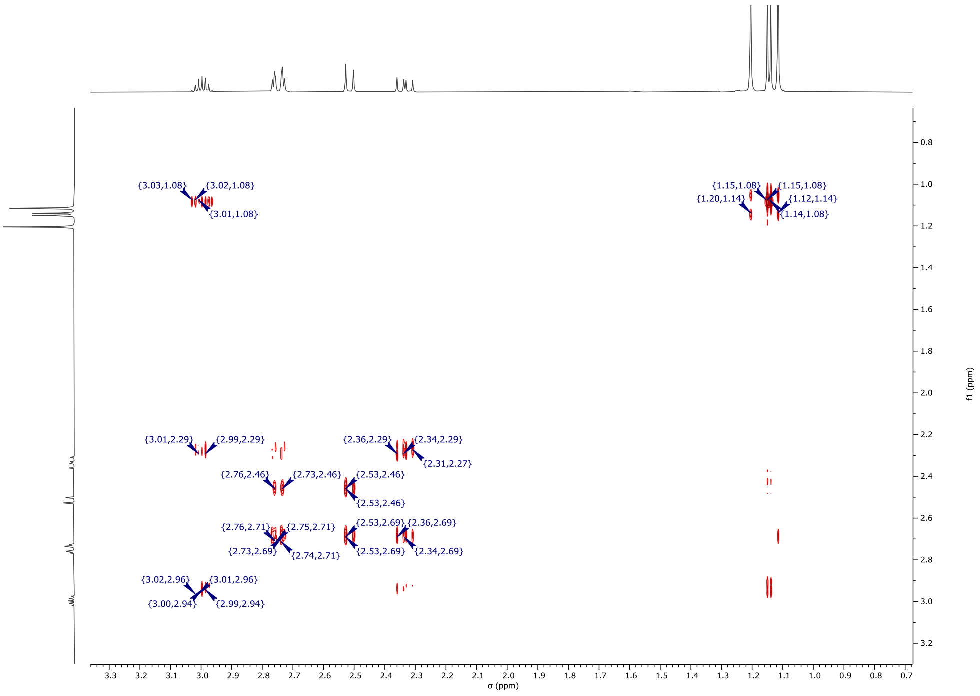

Figure S22. ^{1}H/^{1}H COSY NMR spectrum ($CDCl_3$, 600 MHz, D1 = 25 s) of the isolated levodione.

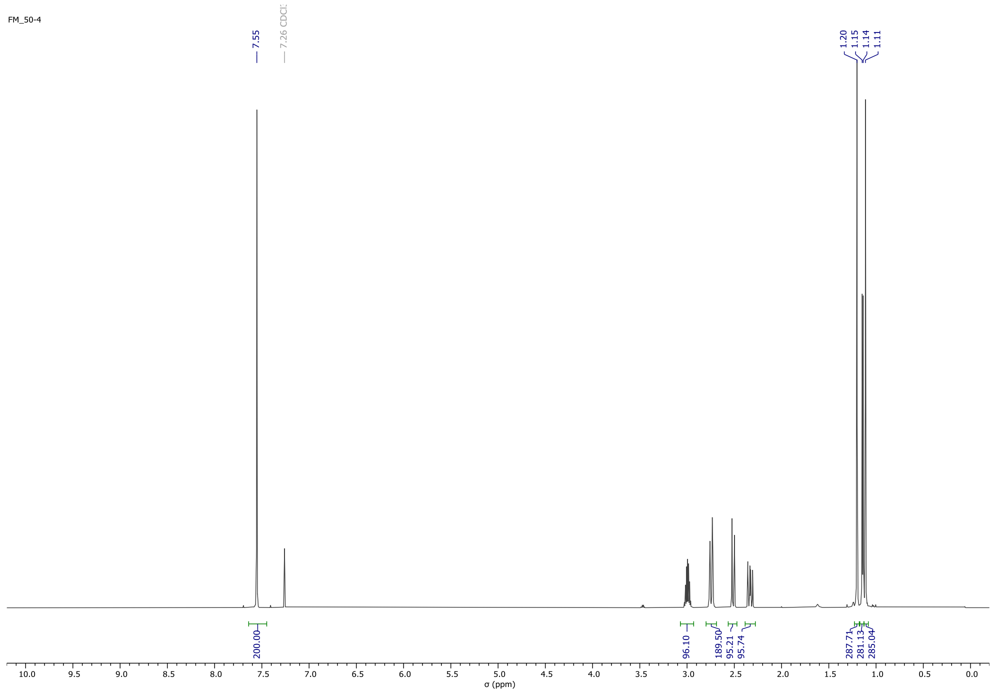

Figure S23. Quantitative ^{1}H NMR spectrum ($CDCl_3$, 600 MHz, D1 = 25 s) of the isolated levodione **4** with 1 equivalent of 1,2,4,5-tetrachlorobenzene as internal standard.

Supplementary References

Al-Shameri, Ammar; Willot, Sébastien J-P; Paul, Caroline E.; Hollmann, Frank; Lauterbach, Lars (2020): H_2 as a fuel for flavin- and H_2O_2-dependent biocatalytic reactions. In *Chem. Commun.* 56 (67), pp. 9667–9670. DOI: 10.1039/D0CC03229H.

Baker, Richard W.; Low, Bee Ting (2014): Gas Separation Membrane Materials: A Perspective. In *Macromolecules* 47 (20), pp. 6999–7013. DOI: 10.1021/ma501488s.

Gonçalves, Leticia C. P.; Mansouri, Hamid R.; PourMehdi, Shadi; Abdellah, Mohamed; Fadiga, Bruna S.; Bastos, Erick L. et al. (2019): Boosting photobioredox catalysis by morpholine electron donors under aerobic conditions. In *Catal. Sci. Technol.* 9 (10), pp. 2682–2688. DOI: 10.1039/C9CY00496C.

Jongkind, Ewald P. J.; Fossey-Jouenne, Aurélie; Mayol, Ombeline; Zaparucha, Anne; Vergne-Vaxelaire, Carine; Paul, Caroline E. (2022): Synthesis of chiral amines via a bi-enzymatic cascade using an ene-reductase and amine dehydrogenase. In *ChemCatChem* 14 (2), Article e202101576. DOI: 10.1002/cctc.202101576.

Joseph Srinivasan, Shiny; Cleary, Sarah E.; Ramirez, Miguel A.; Reeve, Holly A.; Paul, Caroline E.; Vincent, Kylie A. (2021): *E. coli* nickel-iron hydrogenase 1 catalyses non-native reduction of flavins: demonstration for alkene hydrogenation by old yellow enzyme ene-reductases. In *Angew. Chem. Int. Ed. Engl.* 60 (25), pp. 13824–13828. DOI: 10.1002/anie.202101186.

Mifsud, Maria; Gargiulo, Serena; Iborra, Sara; Arends, Isabel W. C. E.; Hollmann, Frank; Corma, Avelino (2014): Photobiocatalytic chemistry of oxidoreductases using water as the electron donor. In *Nat Commun* 5 (1), p. 3145. DOI: 10.1038/ncomms4145.

Nett, Nathalie; Duewel, Sabine; Schmermund, Luca; Benary, Gerrit E.; Ranaghan, Kara; Mulholland, Adrian et al. (2021): A robust and stereocomplementary panel of ene-reductase variants for gram-scale asymmetric hydrogenation. In *Molecular Catalysis* 502, p. 111404. DOI: 10.1016/j.mcat.2021.111404.

Özçam, A. Evren; Efimenko, Kirill; Genzer, Jan (2014): Effect of ultraviolet/ozone treatment on the surface and bulk properties of poly(dimethyl siloxane) and poly(vinylmethyl siloxane) networks. In *Polymer* 55 (14), pp. 3107–3119. DOI: 10.1016/j.polymer.2014.05.027.

Sheldon, Roger A. (2017): The E factor 25 years on: the rise of green chemistry and sustainability. In *Green Chem.* 19 (1), pp. 18–43. DOI: 10.1039/c6gc02157c.

Son, Eun Jin; Lee, Sahng Ha; Kuk, Su Keun; Pesic, Milja; Da Choi, Som; Ko, Jong Wan et al. (2018): Carbon nanotube–graphitic carbon nitride hybrid films for flavoenzyme-catalyzed photoelectrochemical cells. In *Adv Funct Materials* 28 (24), Article 1705232, p. 1705232. DOI: 10.1002/adfm.201705232.

Song, S.-H.; Dick, B.; Penzkofer, A. (2007): Photo-induced reduction of flavin mononucleotide in aqueous solutions. In *Chemical Physics* 332 (1), pp. 55–65. DOI: 10.1016/j.chemphys.2006.11.023.

Tentori, Francesca; Bavaro, Teodora; Brenna, Elisabetta; Colombo, Danilo; Monti, Daniela; Semproli, Riccardo; Ubiali, Daniela (2020): Immobilization of old yellow enzymes via covalent or coordination bonds. In *Catalysts* 10 (2), p. 260. DOI: 10.3390/catal10020260.

Tosstorff, Andreas; Kroner, Cora; Opperman, Diederik J.; Hollmann, Frank; Holtmann, Dirk (2017): Towards electroenzymatic processes involving old yellow enzymes and mediated cofactor regeneration. In *Engineering in life sciences* 17 (1), pp. 71–76. DOI: 10.1002/elsc.201600158.

Villa, Rocio; Ferrer-Carbonell, Claudia; Paul, Caroline E. (2023): Biocatalytic reduction of alkenes in micro-aqueous organic solvent catalysed by an immobilised ene reductase. In *Catal. Sci. Technol.* 13 (19), pp. 5530–5535. DOI: 10.1039/D3CY00541K.

Xiao, Min; Zhou, Jiti; Tan, Yue; Zhang, Aili; Xia, Yuanhua; Ji, Lei (2006): Treatment of highly-concentrated phenol wastewater with an extractive membrane reactor using silicone rubber. In *Desalination* 195 (1-3), pp. 281–293. DOI: 10.1016/j.desal.2005.12.006.
